# Influence of Silver Nanoparticles on the Metabolites of Two Transgenic Soybean Varieties: An NMR-Based Metabolomics Approach

**DOI:** 10.1021/acs.jafc.4c00756

**Published:** 2024-05-15

**Authors:** Amanda L. Quintela, Maria F. C. Santos, Rodrigo F. de Lima, Juliana L. S. Mayer, Gustavo G. Marcheafave, Marco A. Z. Arruda, Cláudio F. Tormena

**Affiliations:** † Physical Organic Chemistry Laboratory, Institute of Chemistry, Universidade Estadual de Campinas, UNICAMP, PO Box 6154, Campinas 13083-970, São Paulo, Brazil; ‡ Laboratory of Plant Anatomy, Institute of Biology, Universidade Estadual de Campinas, UNICAMP, PO Box 6109, Campinas 13083-862, São Paulo, Brazil; § Institute of Chemistry, Universidade Estadual de Campinas, UNICAMP, PO Box 6154, Campinas 13083-970, São Paulo, Brazil; ∥ Spectrometry, Sample Preparation and Mechanization Group, Institute of Chemistry, Universidade Estadual de Campinas, UNICAMP, PO Box 6154, Campinas 13083-970, São Paulo, Brazil

**Keywords:** soybean (Glycine max), silver nanoparticles (AgNPs), metabolomic analysis, nuclear magnetic resonance (NMR), multivariate analysis

## Abstract

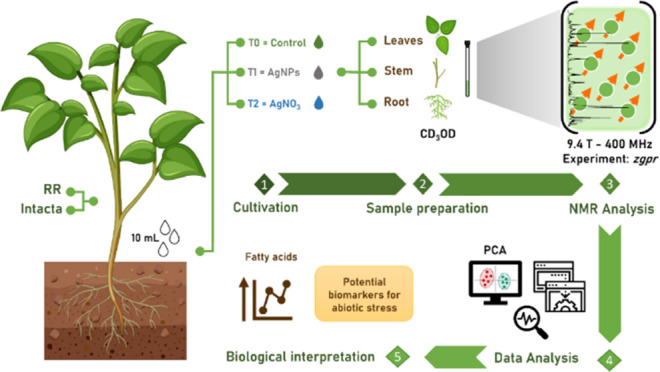

This study investigated the effect of AgNPs and AgNO_3_, at concentrations equivalent, on the production of primary and secondary metabolites on transgenic soybean plants through an NMR-based metabolomics. The plants were cultivated in a germination chamber following three different treatments: T0 (addition of water), T1 (addition of AgNPs), and T2 (addition of AgNO_3_). Physiological characteristics, anatomical analyses through microscopic structures, and metabolic profile studies were carried out to establish the effect of abiotic stress on these parameters in soybean plants. Analysis of the ^1^H NMR spectra revealed the presence of amino acids, organic acids, sugars, and polyphenols. The metabolic profiles of plants with AgNP and AgNO_3_ were qualitatively similar to the metabolic profile of the control group, suggesting that the application of silver does not affect secondary metabolites. From the PCA, it was possible to differentiate the three treatments applied, mainly based on the content of fatty acids, pinitol, choline, and betaine.

## 1 Introduction

Soybean (*Glycine max* (L) Merrill.) is globally significant due to its versatility as a crop, serving as a plentiful source of both protein and oil across various temperate and tropical regions.^1−3^ Soybeans serve multiple purposes, including as a source of oil and food for humans, as well as being utilized as animal feed and biofertilizers for crops.^3^ The diverse applications of soybeans make them a sought-after crop, leading to a rapid rise in demand within the agricultural trade. However, soybean cultivation frequently faces numerous environmental or abiotic stresses, including salinity, drought, high temperatures, heavy metal toxicity, intense light, and nutrient deficiency. These stressors often result in significant losses in both yield and quality of crop production. They can induce various changes at the morphological, physiological, biochemical, or molecular levels within plant cells, ultimately impacting seed germination, growth, and productivity.^3^

The increasing global demand for soybeans and their derivatives has prompted research initiatives that incorporate nanobiotechnology, genome editing, and rapid breeding techniques. These endeavors seek to develop smart cultivars capable of meeting the food security needs of our growing global population.^4^ Developing new genotypes with enhanced yields, increased tolerance to drought and diseases, and shorter growth periods offer significant advantages for agricultural production. Genetic research stands out as a particularly effective approach for furthering the development of high-performance cultivars with desirable agronomic traits, improved nutritional content, and enhanced seed performance.^3,5^ In addition, research has explored the use of nanoparticles (NPs) in agricultural products to improve the nutritional quality of soybeans and offer a better pest control procedure.^7,8^

Nanoparticles (NPs) have significant effects on plant growth, physiology, and molecular responses.^6^ They can spread to various parts of plants, including roots, stems, and leaves, through different pathways, such as stomata, plasmodesmata, xylem, and phloem, as well as transport proteins. In addition, exogenous application of NPs, either to the soil or as a foliar spray, can lead to their absorption through the root epidermis or via apoplastic and symplastic routes to the air surfaces.^7^ Once integrated into plants, NPs trigger the production of reactive oxygen species (ROS).^8^ In response to ROS, plants activate the synthesis of antioxidants, enzymes, and metabolites. This response also involves changes in the physiological and biochemical conditions governing seed germination.^4,8^

Silver nanoparticles (AgNPs) have aroused significant interest among metallic NPs due to their range of properties, including antimicrobial, antifungal, antibacterial, cytotoxic, and phytotoxic effects.^9^ These properties allow them to control and prevent diseases while also affecting the growth and germination of seeds. AgNPs are generally applied in concentrations ranging from 0.5 to 1000 ppm (mg L^–1^), which makes them promising candidates for agricultural applications such as nanopesticides and nanofertilizers.^10,11^ In addition to the performance of NPs for the purposes described above, the interaction of a metal with the plant can generate adverse effects on its development and metabolism, becoming a limiting factor for the introduction of NPs into agricultural practices. Therefore, the study of this topic brings to light the knowledge of the likely harmful or beneficial effects that the use of these materials can have on the food chain.

In this context, metabolomics has emerged as the most promising tool for deciphering the comprehensive metabolite composition within a biological system, for example, in plant species.^12,13^ The application of metabolomics in NPs–organism interaction studies can contribute to the understanding of the compounds generated by this interaction. In several studies, the application of NMR metabolomics has revealed the adaptive responses of soybeans to the use of NPs, identifying the primary and secondary metabolites involved in the adaptation mechanisms.^14−16^ There has been growing interest in identifying, quantifying, and characterizing the diverse range of low molecular weight compounds produced by plants for protection purposes. These compounds serve as biomarkers, indicating biochemical events associated with interactions with NPs.^13^

Given the importance of evaluating the development of soybean crops in the presence of NPs and considering the lack of information on transgenic soybeans, this study reports, for the first time in the literature, the profile of primary and secondary metabolites of two transgenic varieties, RR and Intact, of soybean plants exposed to AgNPs with sizes of approximately 60 nm, investigated through a metabolomics approach based on NMR.

## 2 Materials and Methods

### 2.1 Cultivation of Soybean Plants

The AgNPs and AgNO_3_ solutions used for cultivation were synthesized in a similar way to those used in a previous work.^9^ The AgNPs used have a spherical morphology and an average size of approximately 60 nm.^9^ In the present investigation, metabolic profiles based on ^1^H NMR were analyzed in two cultivars of *Roundup Ready* transgenic soybean seeds (variety 8473RSF – RR Challenge) and Intact transgenic soybean seeds (variety 7166RSF IPRO- Ponta IPRO).^17,18^ The transgenic seeds were kindly donated by the company Brejeiro, city of Nuporanga/São Paulo (Brazil). Soybean seeds were sterilized in 5% (v/v) sodium hypochlorite solution for 5–10 min,^19^ then rinsed thoroughly several times with deionized water. They were germinated and grown in 200 mL plastic pots containing 50 g of a mixture of 50% (v/v) substrate (*BasaPlant*, Brazil) plus 50% (v/v) vermiculite (Vermfloc Agro, Brazil) at pH 6.2. Three seeds were sown per pot at ca. 2 cm deep each. The plants were grown for 21 days in a growth chamber under controlled temperature (27 ± 0.1 °C) and photoperiod (12 h).

The plants cultivated in the same experiments formed 6 groups, each with 10 samples (analytical replicates), thus resulting, at the end of each cultivation, in 10 samples per group: RR-control (T0-RR), Intact-control (T0-I), RR-AgNPs (T1-RR), Intact-AgNPs (T1-I), RR-AgNO_3_ (T2-RR), and Intact-AgNO_3_ (T2-I). Each plant was measured in height and weight in the three compartments. During the first 7 days, all plants were irrigated with deionized water only. During the following 14 days, between the 8th and 21st days of cultivation, the plants were irrigated with Ultrapure water, and 10 mL of solutions containing AgNPs or AgNO_3_ were added daily to the respective groups. On the 22nd day, at the end of cultivation, each plant that was treated with AgNPs or AgNO_3_ received the same amount of Ag (2.5 mg) in the soil, resulting in a concentration of 50 mg kg^–1^ Ag in the substrate to evaluate possible changes in the profile metabolomics of soybean plants. Then, the compartments of each plant were separated into roots, stems, and leaves, which were immediately frozen in liquid nitrogen, freeze-dried for 2 days, and macerated using a batch analytical mill, resulting in the formation of a powder sample.

### 2.2 Light and Fluorescence Microscopy

With the intention of discovering how silver in nanoparticle and ionic form can affect the microscopic structure of the plant, an anatomical analysis was carried out. For anatomical analyses, three leaf, stem, and root samples from each variety studied were used. Samples were fixed in neutral buffered Formalin^20^ for 48 h and then stored in a 70% (v/v) ethanolic solution. Part of the samples were dehydrated until they reached 100% alcohol and then infiltrated with hydroxy-ethyl-methacrylate (*Leica Historesin*) according to the manufacturer’s instructions. The blocks obtained after the infiltration process were sectioned using a rotating microtome in the longitudinal and transverse planes, with a thickness between 5 and 10 μm. The sections obtained were stained with 0.05% (v/v) toluidine blue in phosphate buffer and citric acid with a pH of 4.52^21^ for structural analysis. For the detection of different chemical groups, the sections were subjected to the following dyes and reagents: Sudan IV^22^ and Sudan Black B^23^ for total lipids, ruthenium red for acid mucilage,^24^ Lugol for starch grains,^25^ and ferric chloride for phenolic compounds.^25^ Sections stained with Calcofluor White^25^ and unstained were analyzed using a DAPI autofluorescence filter (example: BP 350–365 nm, in 445–450 nm). After staining, the stained sections were mounted on a slide and covered with a coverslip using Entellan synthetic resin. The results were documented by capturing images with an Olympus DP71 video camera coupled to an Olympus BX 51 microscope.

### 2.3 Scanning Electron Microscopy

Samples of leaf, stem, and root organs were subjected to the critical point drying method^26^ using the CPD-030 Critical Point equipment. They were then mounted on aluminum supports and coated with a thin layer of 30–40 nm gold using the Sputter Coater SCD-050 equipment. Electromicrograph analyses were carried out using the JSM 5800LV Scanning Electron Microscope, located at the Electron Microscopy Laboratory (LME) at the Institute of Biology at the UNICAMP.

### 2.4 Acquisition of the NMR Spectra

Sample preparation consisted of separating plant organs into roots, stems, and leaves. As there were many samples to be analyzed, it was decided to perform direct extraction, using deuterated solvent^27^ as an extraction solvent, thus reducing the steps and analysis time, in accordance with the methodology proposed by Beckonert et al.^28^ with some modifications. For NMR analyses, 0.05 g of each freeze-dried soybean leaf sample was dissolved in 1.0 mL of CD_3_OD containing 0.05% TMS. For the stem, 0.04 g was weighed, and for the roots, 0.03 g was weighed. Extracts were prepared in triplicates and homogenized for 1 min in a vortex. The extracts were placed in a *cup-horn* sonicator,^29^ and the program consisted of 70% amplitude for 1 min, with 30 s intervals (on/off). After extraction, the samples were centrifuged for 5 min at 12,000 rpm. A 600 μL aliquot of the supernatant was transferred to the 5 mm NMR tube.

^1^H NMR spectra were acquired on a Bruker Avance III NMR spectrometer, operating at 9.4 T, observing the ^1^H nucleus at 400 MHz and the ^13^C nucleus at 100 MHz, equipped with an automatic sample changer, probe 5 mm BBI (broadband inverse detection) with ATMA (automatic tunning matching adjustment). The ^1^H NMR experiments were carried out at 298 K. 2D NMR spectra were acquired on a Bruker Avance III NMR spectrometer, operating at 14.1 T, observing the ^1^H nucleus at 600 MHz and the ^13^C nucleus at 150 MHz, equipped with a probe 5 mm TBI (triple resonance broadband inverse detection). Due to the characteristics of the samples under study, it was necessary to acquire ^1^H NMR spectra with suppression of the solvent signal. Analyses in methanol were performed using the simplest presaturation pulse sequence, the *zgpr* saturating residual OH signal at 4.88 ppm, with 64 scans, each FID was acquired using 64 k data points, 20.0 ppm for spectral window (SW), leading to 4.08 s for acquisition time (AQ). It is worth mentioning that in this evaluation, extractions were carried out in nine replicates for the three treatments of the two varieties, totaling 54 spectra acquired for each compartment of the plant.

The TopSpin 3.6.5 software was used for NMR data acquisition, processing, and analysis, using IconNMR module for controlling the automation (locking, tuning, matching, and shimming) for spectra acquisition. Phase and baseline corrections were also performed using the TopSpin 3.6.5 software. All spectra were referenced to TMS (δ_H_ 0.0 ppm). The multiplicities observed were labeled as s (singlet), d (doublet), t (triplet), dd (doublet of doublets), q (quartet), m (multiplet), and br (broad singlet). For the structural identification of metabolites from leaf, stem, and root extracts, all assignments were based on the analysis of 1D and 2D homo- and heteronuclear NMR experiments (^1^H–^1^H COSY, ^1^H–^13^C HSQC-TOCSY, ^1^H–^13^C multiplicity-edited HSQC, and ^1^H–^13^C HMBC), along with the use of the public database of NMR, public NMR databases such as HMDB and literature data.^2,14,29−34^

### 2.5 Statistical Analysis

To calculate the significant differences between the three treatments, one-way ANOVA and Tukey’s test from Microsoft Excel for Microsoft 365 MSO (Version 2401 Build 16.0.17231.20236) 64 bits were used to assemble the spreadsheets and construct the plant evaluation graphs. Principal component analysis (PCA) was performed to investigate the variability of primary and secondary metabolites of soybean plants subjected to three different treatments. ^1^H NMR spectra were automatically reduced to the ASCII extension of the AMIX program (version 3.9, Bruker BioSpin) to perform principal component analysis (PCA). The signal intensities of the spectra, in the region of δ_H_ 0.5–10.5 ppm, were scaled based on the TMS signal intensity, and the regions were integrated and divided into the bucket of 0.04 ppm. Areas referring to the residual OH signal from solvent (δ_H_ 4.5–5.0 ppm) and the residual CHD_2_OH group from solvent (δ_H_ 3.30–3.33 ppm) signals were excluded from the data matrix. The scores and loading graphs were analyzed to discriminate the types of treatments and varieties of transgenic soybean plants.

## 3 Results and Discussion

### 3.1 Assessment of Plants after Cultivation

**Figure 1:**
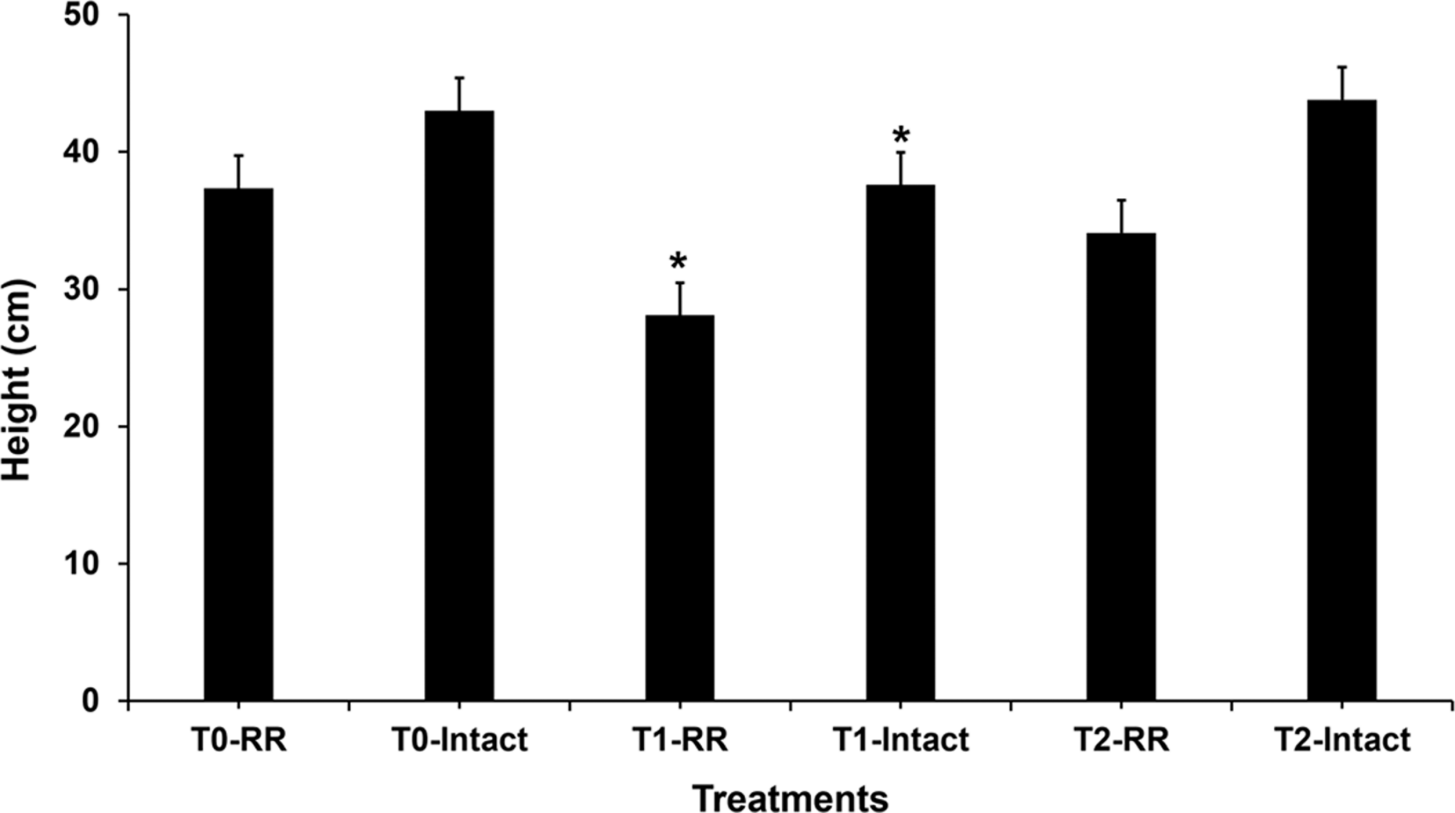
Height of transgenic soybean plants (*n* = 10 for each group) after 21 days of cultivation at a concentration of 50 mg kg^–1^. Significant changes (*p* < 0.5) are signed with (*).

**Figure 2:**
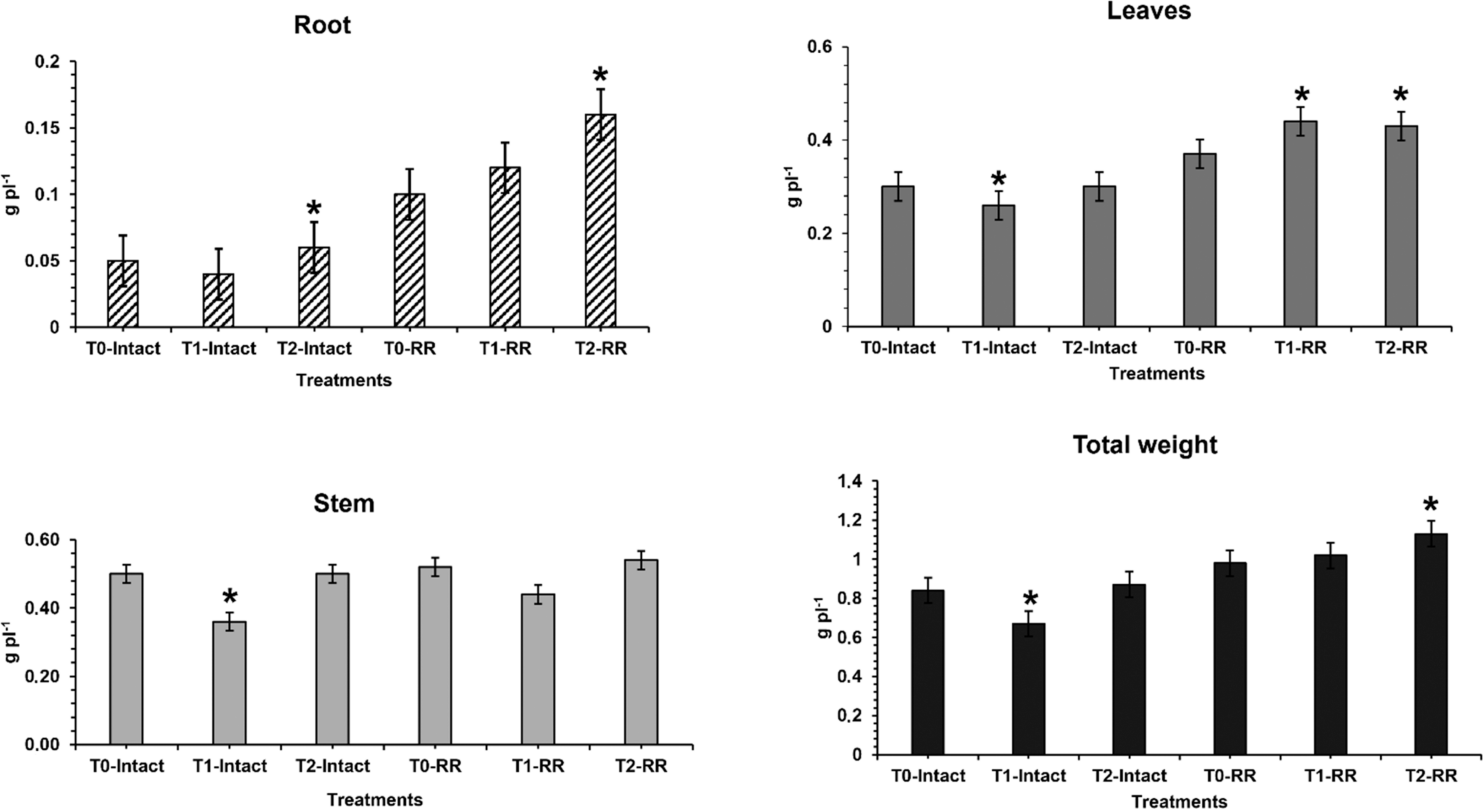
Dry weight of transgenic soybean plants (*n* = 10 for each group) after 21 days of cultivation at a concentration of 50 mg kg^–1^. Significant changes (*p* < 0.5) are signed with (*).

After planting, the length of the stems (Figure 1) and dry weight (relative mass) of the plants in each group were evaluated (Figure 2). In terms of stem height, it was observed that the RR and Intact plants treated with AgNPs (T1 treatment) showed a decrease of 25.07 and 12.56%, respectively, compared to the plants in the control group. For the group treated with AgNO_3_ (T2 treatment), there was no statistically significant difference in terms of stem length when compared to the control groups, which suggests that ion Ag^+^ is not affecting the soybean growth under the applied cultivation conditions. In terms of size, the literature reports that AgNPs ranging from 20 to 80 nm stunt growth;^35^ however, in our work, only the T1 treatment for both varieties significantly changed (Tukey’s test – *p* < 0.5) the plants in terms of height, when compared to the control group, while phytotoxicity is dependent on the concentration and size of NPs.^35^ According to Figure 2, it is clear that both the addition of AgNPs and AgNO_3_ resulted in significant changes in the total mass (dry weight) of the soybean plants when comparing the Intact and RR groups with the control group.

The toxic effects of AgNPs in plants are related to the absorption, distribution, and translocation of NPs within the plant and are attributed to the accumulation of reactive oxygen species (ROS), which induce cell membrane rupture and oxidative damage. However, it is still unclear to what extent AgNP toxicity results from the nanoparticulate form and how much toxicity is related to the released silver ions (Ag^+^).^35,36^ Although the exact mechanism of how they affect plant growth has not yet been fully elucidated, studies have shown that, after absorption, AgNPs can accumulate and deposit in the plant cell wall, in the intermembrane space, and inside the cell. Changes in germination rate, fresh and dry weight, and root and shoot length are frequently observed markers of AgNPs-induced^37^ phytotoxicity. And since plants are primary producers in any ecosystem, the bioaccumulation of NPs in plants can lead to their participation within food chains.

Our results indicate that treatment with 60 nm AgNPs (T1 group) and AgNO_3_ (T2 group) does not seem to affect the morphology and physiology of soybean leaves because no qualitative observations of scratches or dark spots were observed. However, light brown spots were observed on the roots and stems, indicating that tissue necrosis occurred when the roots were exposed to AgNPs and AgNO_3_, proving that Ag caused toxic effects at a macroscopic level, given that there was less root development and the appearance of necrotic areas in the basal part of the stems, as well as the appearance of areas with some damage or chlorosis in the leaves of groups T1 and T2.^1^ This color occurs due to the adsorption of AgNPs alone or together with cell wall materials or secondary metabolites produced by the root tips.^16^ Comparing the response of roots and shoots, all treatments tested induced a stronger prominent effect on roots, corroborating a much greater accumulation of AgNPs in roots than in leaves.^1^ A study carried out with tobacco plants (*Nicotiana tabacum*) grown in vitro^37^ involved exposure to AgNPs, as well as ionic silver (AgNO_3_), applied in the same concentrations, alone or in combination with cysteine, a strong silver ligand, reported a greater accumulation of Ag^+^ in roots and leaves after exposure to AgNPs compared to AgNO_3_. This was correlated with a predominantly greater impact of silver in NP than silver in ionic form on oxidative stress parameters, although no serious damage to important biomolecules was observed.^37^

As reported by Galazzi et al.,^1^ nontransgenic and transgenic soybean plants cultivated with AgNPs and AgNO_3_ accumulate a significant amount of silver in the aerial parts (stem and leaves). The translocation rate for RR soybean plants with AgNPs and AgNO_3_ was around 100 and 65%, respectively, of the Ag^+^ assimilated by the roots that were translocated to the aerial parts. This study suggests that transgenic soybean roots assimilate Ag^+^ during exposure to nanoparticles.^1^ These results may be due to the application method (directly to the soil), where, in both cases, the roots came into direct contact with the exposure medium. Regarding the performance of AgNPs, it was expected that after penetrating the roots, they would be able to penetrate the cell wall and plasma membranes of the epidermal layer of roots and vascular tissues.^38^ However, in this study, the average size of AgNPs used (60 nm) made translocation to the aerial part difficult, which can be corroborated by the study by Chacón-Madrid et al.,^38^ in which there was low Ag translocation (around 0.2% only) to the aerial compartments. This can be explained by the fact that AgNPs tend to accumulate mainly in the apoplast of root cells, with only a small amount being transported to other tissues, in addition to the fact that the soybean plant is dicotyledonous, which has difficulty in transporting Ag.^39^ The results indicate that for different plant organs, specific responses occur to deal with oxidative stress induced by Ag.^37^

Another hypothesis is that this result may be associated with the coated surface of the nanoparticle. In this case, the AgNPs used in this work are stabilized with citrate, and their mechanism is based on charge repulsion.^40^ As a main observation, AgNPs coated with citrate showed the greatest tendency toward instability as an aggregation process. The same AgNP-coating behavior was discussed by Košpić et al.^37^ during the investigation of the uptake of AgNPs by the tobacco plant. Therefore, understanding this behavior related to nanoparticle coating can support experiments involving nanoparticles and biological systems. However, the study carried out by Ma et al.^41^ showed that AgNPs up to 40 nm can be absorbed by the roots of *Arabidopsis thaliana* and transported to the shoot. Furthermore, confocal/multiphoton microscope observation showed that AgNPs were accumulated in the columella in roots.^8^

### 3.2 Microscopy

The results of scanning electron micrographs of the leaf surface of the two varieties studied revealed the presence of epidermal cells juxtaposed with elongated nonglandular trichomes on both sides of the epidermis. The plants subjected to treatments with AgNPs showed the occurrence of small areas with adaxial surface cracks in the Intact variety (Figure S1), whereas in the RR variety, glandular trichomes fell. Treatment with AgNO_3_ resulted in the occurrence of cracks and the loss of trichomes on both sides of the epidermis of the two varieties analyzed, with the greatest impact observed in the Intact variety, which showed greater susceptibility. In the leaves that were subjected to treatments with AgNO_3_, a process of cell autolysis was observed with degradation of the cell wall in the parenchyma cells located close to the abaxial surface in the central vein. Treatment with AgNPs did not cause any damage or changes in the structure of the central vein and leaf mesophyll, and this may be due to the low translocation factor of nanoparticles from the root to the aerial part of the plant, as already reported in the work of Galazzi et al.^17^Figure S1 shows scanning electron micrographs in the leaf compartment for RR and Intact soybean plants.

On the stem surface, using scanning microscopy, it was possible to observe elongated epidermal cells with the presence of glandular and nonglandular trichomes in both varieties. In treatment T1, a drop in trichomes was observed, in addition to the random appearance of small lesions in the epidermal cells in all varieties (Figure S2c). Plants subjected to T2 treatment showed damage to epidermal cells, evidenced by significant cracks (Figure S2e,f), especially in the transition region between the stem and root tissue, known as the neck. The collapse of damaged cells toward the center of the organ was also observed. These damaged cells had little cellulose in their walls, indicating cell death.

In both T1 and T2 treatments, loosening of the periclinal and anticlinal walls of the epidermal cells and cortical parenchyma of the roots was observed. In the Intact variety, which showed greater susceptibility, the formation of tyloses was observed in the vessel elements close to the most affected regions. In addition, phenolic compounds were detected in the damaged cells as well as in the stem region (see Figures S3i,l and S4d,e). However, in the treatment with AgNPs, the damage was restricted to the epidermal cells and the first layers of subepidermal parenchyma (Figures S3e,h,k and S4b), while in the treatment with AgNO_3_, the damage affected the entire cortical region, reaching the vascular cylinder (Figures S3f,i,l and S4c). In the control treatment, epidermal and parenchymal cells did not show loosening of the cell walls or the accumulation of phenolic compounds in and near damaged cells (Figures S3d,g,j and S4a). In fluorescence analyses using Calcofluor White, it was found that damaged cells had little cellulose in their walls (Figure S4g–i) when compared to the control treatment (Figure S4f), suggesting that loosening can lead to cell death (Supporting Information). The root was the most affected compartment for both treatments, in which the presence of cracks was diagnosed, similar to those that occurred in the stem region; however, it is already reported in the literature that AgNPs damage epidermal cells more when compared with AgNO_3_.^37^

### 3.3 Identification and Comparison of Metabolite Profiles of the Transgenic Soybean Plants

**Figure 3:**
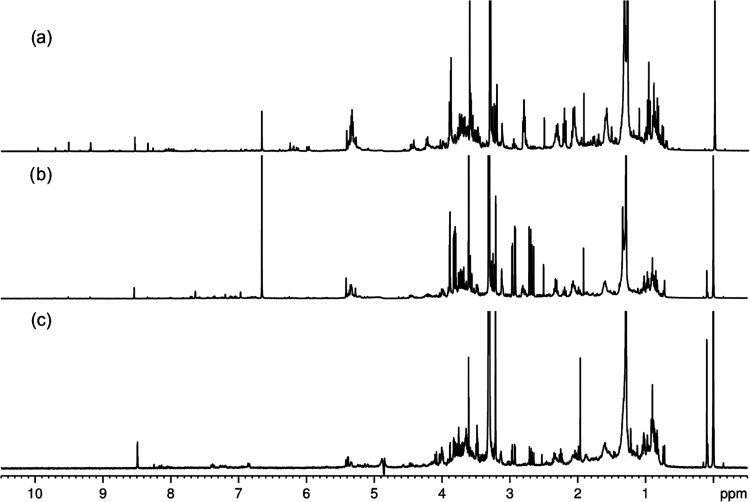
^1^H NMR spectra (400 MHz, CD_3_OD) for (**a**) leave, (**b**) stem and (**c**) root samples of soybean for the control group.

^1^H NMR analysis was used to investigate the qualitative changes in primary and secondary metabolites present in soybean leaves, stems, and roots subjected to treatments with AgNPs and AgNO_3_. Most of the identified compounds have already been reported in the literature and are involved in the primary metabolism of carbon and nitrogen, including carbohydrates, organic acids from the TCA cycle, and amino acids.^32^ Comparison of ^1^H NMR spectra did not reveal many or almost no qualitative differences in the metabolites for CD_3_OD extraction from the three plant compartments: leaves, stems, and roots, although the TMS signal (δ_H_ 0.0 ppm) is more intense in spectrum C due to the low concentration of the root sample, as shown in Figure 3. Furthermore, the ^1^H NMR spectra of the two varieties are qualitatively similar. Table 1 describes the metabolites and chemical shifts assigned to the ^1^H NMR spectra of the CD_3_OD extracts for leaves, stems, and roots. The assignments were based on the literature^2,32,33,42,43^ and supported by 2D NMR spectra. All 2D NMR spectra and chemical structures can be found in the Supporting Information (Figures S5–S18).

**Table 1:** Metabolite and Chemical Shift Assignment in ^1^H NMR Spectra (600 MHz) of CD_3_OD Extracts for Leaves, Stems, and Roots of Transgenic Soybean^a^

metabolites	δ ^1^H (multiplicity, *J* in Hz)	δ ^13^C (HSQC/HMBC)	leaves	stems	roots	references
fatty acids						
(1) saturated fatty acids	0.73 (t, 2.93)	12.2	√	√	√	(29,30)
(2) oleic acid (ω-9)	0.84 (d, 6.60); 1.34–1.28 (m); 1.60 (q, 6.52); 2.07 (t, 6.80); 2.3 (m); 5.40–5.30 (m)	23.2; 24.1; 26.1; 28.7; 35.4; 130.5	√	√	√	(29,30)
(3) linoleic acid (ω-6)	0.90 (t, 6.90); 1.34–1.28 (m); 1.6 (q, 6.52); 2.07 (t, 6.80); 2.31 (m); 2.77 (t, 6.17); 5.4–5.30 (m)	14.9; 24.1; 26.1; 28.7; 35.4; 27.1; 130.5	√	√	√	(29,30)
(4) α-linolenic acid (ω-3)	0.97 (t, 7.56); 1.34–1.28 (m); 1.60 (q, 6.52); 2.07 (t, 6.80); 2.31 (m); 2.81 (t, 6.40); 5.40–5.30 (m)	14.9; 24.1; 26.1; 28.7; 35.4; 27.1; 130.5	√	√	√	(29,30)
(5) α-glycerol	4.21 (m); 4.43 (m)	64.6	√	x	x	(30)
(6) β-glycerol	5.25 (m)	72.3	√	x	x	(30)
amino acids						
(7) valine	0.77 (d, 6.65); 1.74 (m); 3.47 (m)	20.6; 28.2; 66.4	√	√	√	(14,32)
(8) isoleucine	0.84 (d, 6.60); 0.87 (br); 1.09 (m); 1.26 (m); 3.47 (m)	12.9; 12.8; 25.9; 34.6; 66.4	√	√	√	(14,32)
(9) leucine	1.01 (br); 1.02 (br); 1.17 (m); 1.61 (m); 4.03 (m)	20.4; 22.4; 38.8; 23.35; 52.3	√	√	√	(14,32)
(10) lysine	1.18 (m); 1.51 (br); 1.60 (m); 2.19 (m); 3.08 (m)	26.1; 22.3; 30.8; 38.3; 58.1	√	√	√	(14,32)
(11) threonine	1.22 (m); 3.50 (m), 4.22 (m)	18.6; 71.8; 70.6; 174.0	√	√	x	(32)
(12) alanine	1.46 (d, 7.20); 4.03 (m)	17.9; 52.3; 175.4	√	√	x	(2,31,30)
(13) arginine	1.60 (m); 1.85 (t, 6.72); 3.34 (m); 3.79 (m)	30.7; 25.0; 54.8; 57.0	√	√	x	(14)
(14) glutamic acid	1.82 (m); 2.23 (m); 3.61 (m)	25.3; 32.0; 53.5	√	√	x	(14)
(15) γ-aminobutyrate (GABA)	1.86 (t, 6.74); 1.96 (m), 2.08 (m)	25.2; 12.8; 21.5	√	√	x	(14,32)
(16) glutamine	2.03 (m); 2.07 (m); 2.18 (m); 3.83 (m)	33.0; 29.0; 53.0	√	√	√	(14)
(17) aspartic acid or (18) asparagine	2.69 (dd, 16.90, 9.30); 2.94 (dd, 16.95, 3.35); 3.81 (dd, 9.30, 3.45)	36.5; 53.7	x	√	√	(2,32)
(19) tryptophan	3.05 (m); 3.90 (m); 7.04 (t, 7.40); 7.12 (t, 7.40); 7.19 (s); 7.36 (d, 8.12); 7.70 (d, 8.12)	39.4; 53.0; 120.9; 123.6; 125.5; 120.0; 120.0; 171.6	√	√	x	(2,32)
(20) tyrosine	3.05 (m); 3.90 (m); 6.72 (m); 7.19 (m)	39.4; 53.0; 116.3; 125.5; 171.6	√	√	x	(2,32)
(21) histidine	3.25 (m); 3.61 (m); 6.8 (s); 7.97 (s)	27.1; 53.5; 117.1; 132.2	√	x	x	(2)
(22) glycine	3.46(m)	41.17	√	√	√	(31)
(23) betaine	3.90 (s); 4.37 (m)	53.2; 62.7	√	x	x	(34)
organic acids						
(24) lactic acid	1.48 (d, 6.66); 3.40	29.7; 74.0	√	x	x	(31,32)
(25) acetate	1.96 (s)	13.1	√	√	√	(2,32)
(26) citric acid	2.22 (m); 2.33 (m)	43.6; 43.6	√	√	x	(14)
(27) malic acid	2.38 (m); 2.58 (m); 4.44 (t, 3.36)	31.4; 51.2	√	√	x	(14)
(28) succinic acid	2.50 (s)	33.4	√	√	x	(2,32)
(29) pinitol	3.60 (s)	74.9	√	x	x	(31,32)
(30) fumaric acid	6.65 (s)	135.5	√	√	x	(31,32)
(31) gallic acid	6.89 (s)	117.0	√	√	x	(34)
sugars						
(32) frutose	4.23 (d, 7.6)	106.0	√	x	x	(14)
(33) β-glucose	3.47 (H6, d, 2.15); 3.69 (H4, H5, m); 3.73 (H3, dd, 5.28, 2.88); 3.82 (H2, dd, 3.35, 0.90); 4.56 (d, 7.8)	69.1 (C6); 73.0 (C5); 72.5 (C4); 72.2 (C3); 73.1 (C2); 98.4 (C1)	√	√	x	(14)
(34) α-glucose	3.47 (H6, d, 2.15); 3.69 (H4, H5, m); 3.73 (H3, dd, 5.28, 2.88); 3.82 (H2, dd, 3.35, 0.90); 5.10 (d, 3.7)	69.1 (C6); 73.0 (C5); 72.5 (C4); 72.2 (C3); 73.1 (C2); 94.3 (C1)	x	√	x	(14)
isoflavones						
(35) genistein	6.25 (H6, d, 1.68); 6.68 (H8, d, 2.96); 6.80 (H 3′,5′, d, 8.24 Hz); 7.36 (H2′, 6′, d, 8.15); 8.17 (H2, s)	103.8 (C6); 117.2 (C3′, 5′); 110.6 (C2′, 6′)	√	√	x	(33,30)
(36) glycitin	6.36 (H5, d, 3.12); 7.02 (H8, d, 1.20); 5.48 (m, anomeric); 3.82–3.47 (other signals of glucose); 3.71 (OCH_3_)	110.3 (C5); 101.9 (C1”); 56.0 (C6)	x	√	x	(33,30)
(37) glycitein	6.80 (H 3′,5′, d, 8.24); 6.35 (H8, d, 1.64); 6.74 (H5, d, 1.24); 7.45 (H2′, 6′, d, 8.68); 8.17 (H2, s); 3.71 (OCH_3_)	117.6 (C3′, 5′); 129.5 (C2′, 6′); 56.0 (C6)	√	√	x	(33,30)
(38) genistin	6.80 (H 3′,5′, d, 8.24); 6.89 (H6, d, 2.20); 7.32 (H8, d, 2.68); 7.36 (H2′, 6′, d, 8.15) 8.17 (H2, s); 5.48 (m, anomeric); 3.82–3.47 (other signals of glucose)	117.2 (C3′, 5′); 117.0 (C6); 110.7 (C2′, 6′); 101.9 (C1”)	x	√	x	(33,30)
(39) daidzin	6.80 (H 3′,5′, d, 8.24); 6.91 (H6, dd, 8.96, 2.20); 7.41 (H2′, 6′, d, 8.95); 7.71 (H8, d, 2.76); 8.27 (H2, s); 8.75 (H5, d, 8.76); 5.48 (m, anomeric); 3.82–3.47 (other signals of glucose)	117.2 (C3′, 5′); 117.1 (C6); 129.5 (C2′, 6′); 101.9 (C1”)	x	√	x	(14,30)
(40) daidzein	6.80 (H 3′,5′, d, 8.24); 6.91 (H6, dd, 8.96, 2.20); 6.92 (H8, d, 2.20); 7.45 (H 2′, 6′, d, 8.95); 8.08 (H5, d, 9.28); 8.26 (H2, s)	117.2 (C3′,5′); 117.1 (C6,8); 129.5 (C2′,6′)	√	√	x	(14,30)
other compounds						
(41) choline	3.20 (m); 3.72 (m); 3.99 (t, 5.50)	54.8; 62.5; 57.2	√	x	x	(2,30−31,32)
(42) phosphorylcholine	3.20 (m); 3.24 (s); 3.48 (t, 4.90); 3.74 (dd, 9.75, 2.50)	52.8; 85.2; 67.4; 60.8	√	x	√	(34)
(43) glycerophosphorylcholine	3.64 (m); 3.67 (m); 4.24 (m); 4.45 (m)	71.2; 68.3; 70.4; 68.6	x	√	√	(34)
(44) trigonelline	8.06 (s); 8.86 (s); 8.98 (s); 9.21 (s)	128.5; 147.01; 126.5; 147.5	√	x	x	(31)

a Multiplicity: s (singlet), d (doublet), t (triplet), dd (doublet of doublets), q (quartet), m (multiplet), and br (broad singlet). √, present on compartments; x, does not present on compartments. ^13^C data were assigned by (^1^H–^13^C) HSQC edited multiplicity and (^1^H–^13^C) HMBC experiments.

### 3.4 Comparison of Leaves, Stems, and Roots Metabolite Profiles of Transgenic Soybean Varieties

**Figure 4:**
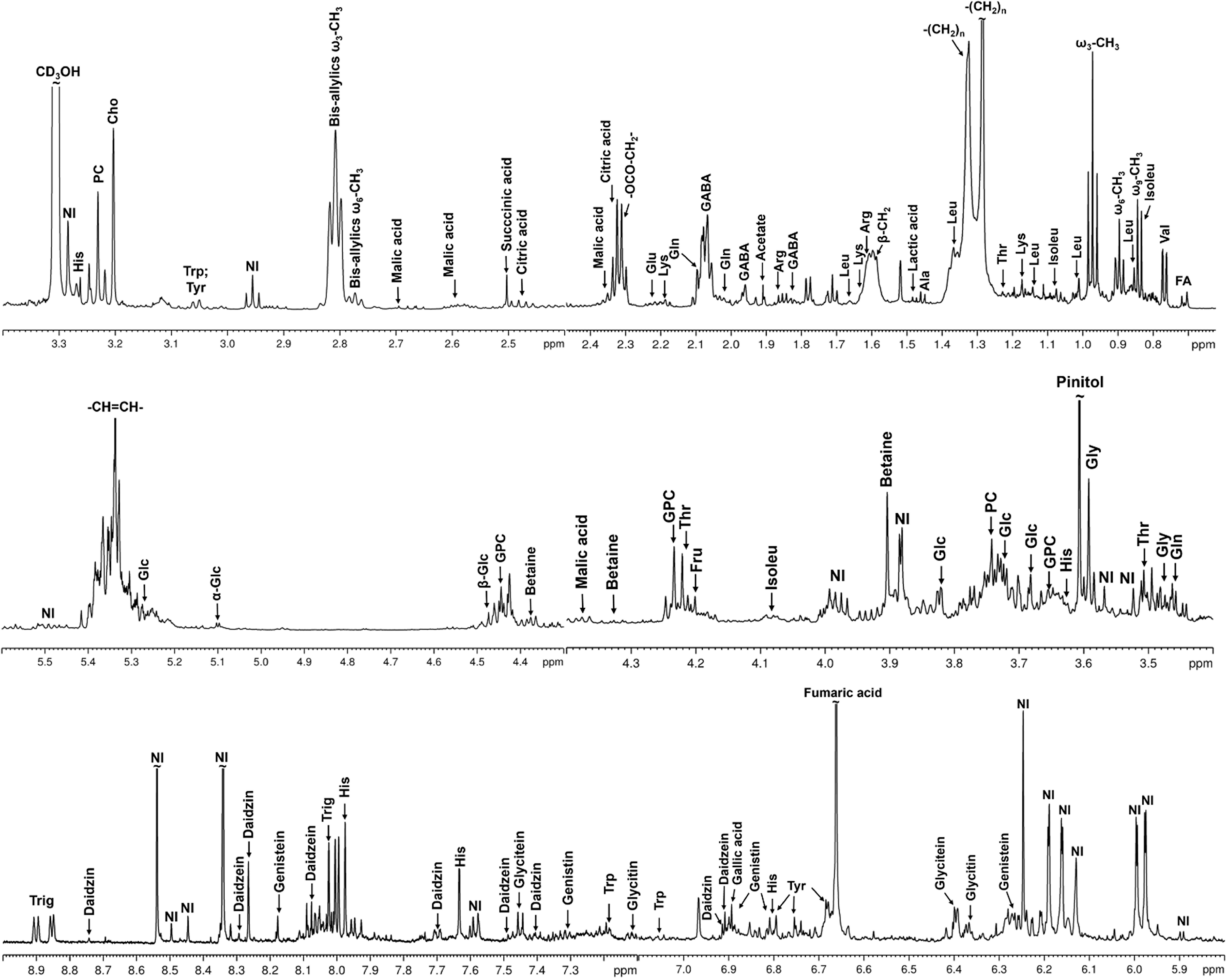
Expansions of ^1^H NMR spectrum (600 MHz) of CD_3_OD extracts for soybean leaves from the control group. Abbreviations: Ala, alanine; Arg, arginine; Cho, choline; FA, fatty acids; Fru, fructose; GABA, 4-aminobutyrate; Glc, glucose; Gln, glutamine; Glu, glutamic acid; Gly, glycine; GPC, glycerophosphorylcholine; His, histidine; Isoleu, isoleucine; Leu, leucine; Lys, lysine; NI, nonidentified; Trp, tryptophan; Thr, threonine; Trig, trigonelline; Tyr, tyrosine; and Val, valine.

The identification of metabolites extracted from the leaves, stems, and roots of transgenic soybean plants, both Intact and RR, in deuterated methanol was carried out by evaluating the ^1^H NMR spectrum. The signals obtained were compared with the spectra of naturally occurring substances in soybean plants obtained under the same experimental conditions as the leaf samples.^2,32^Figure 4 shows the ^1^H NMR spectrum of the leaves, which makes it easier to visualize the compounds that have been identified so far. In a general and simplified way, the ^1^H NMR spectrum can be divided into regions in which a certain class of substance is detected through the chemical shifts and multiplicity of hydrogen signals that characterize them.

In general, the region of the spectrum from δ_H_ 0.5 to 4.5 ppm is characteristic of aliphatic hydrogens. Between δ_H_ 0.70 and 0.95 ppm, methyl signals are observed for different saturated and unsaturated fatty acids. As seen in Table 1, the triplet at δ_H_ 0.97 ppm refers to the methyl group (−CH_3_) of α-linolenic acid, in addition to other overlapping signals in smaller chemical shifts that correspond to the methyl groups of other fatty acids present in soybean plants.^44^ The signals observed in the region of δ_H_ 1.90–2.0 ppm correspond to the methylene hydrogens of linolenic (18:3), linoleic (18:2), and oleic (18:1) acids.^44^ The presence of fatty acids occurs because during senescence, the period of plant aging, the metabolism of lipids in the leaf’s plasma membrane undergoes a degradation in the structural and functional integrity of cell membranes,^32^ which are particularly important in the plant’s defense against fungal and bacterial pathogens, which directly attack the membrane that covers the leaf.^45,46^ The most intense signs of linolenic acid may be associated with the plant’s defense system against rain and humidity. Excess moisture makes the plant more susceptible to fungal attack. Because of this, the plant increases the production of fatty acids such as linolenic acid to waterproof its leaves and protect itself from fungi.^47^ The presence of characteristic signs of the amino acid GABA (γ-aminobutyric acid) was verified and considered in plants to be an important metabolite for mitigating stress caused by environmental or abiotic factors.^32^ In addition, other amino acids were also identified, such as alanine, arginine, betaine, and glycine (Table 1). Most physiological processes are regulated by amino acids and derivatives. Choline, synthesized by the metabolism of glycerophospholipids, is a vital metabolite in eukaryotes as it is necessary to synthesize phosphatidylcholine (lecithin), a constituent of the cell membrane.^46^ Furthermore, chloroplast enzymes catalyze the two-step oxidation of choline to glycine betaine (GlyBet), a plant osmoprotectant, and accumulates in response to abiotic stresses and is responsible for protective mechanisms during early plant leaf development of soybean.^14^ It has already been reported that there is a close association between choline and fatty acids in iron-deficient *G. max* leaves.^2^

Characteristic signals of aspartic acid and asparagine at δ_H_ 2.69, 2.94, and 3.81 ppm are more concentrated and resolved in extracts from stem and root samples and then in the leaf compartment. It can be inferred that in transgenic soybean plants treated with Ag, these substances were best identified only in the stem and root compartments, which can be considered unprecedented since what is reported in the literature is that these compounds were identified only in the leaves.^2,14^

Signals in the carbohydrate region are highly clustered and overlapping. This region is characteristic of signals from the anomeric hydrogens of fructose (δ_H_ 4.23 ppm), β-glucose (δ_H_ 4.56 ppm), and α-glucose (δ_H_ 5.10 ppm). The singlet at δ_H_ 3.6 ppm corresponds to pinitol, an alcohol common in legumes and has been described as an osmoprotector,^14^ associated with an adaptation mechanism to reduce damage during periods of high temperatures and to withstand osmotic stress, such as during rain seasons, water deficit, and drought.^46^

In the central region are the characteristic signals of olefinic hydrogens, −CH=CH–, of unsaturated fatty acids, which appear in the region of δ_H_ 5.30–5.40 ppm.^42^ In the aromatic region of the ^1^H NMR spectrum, δ_H_ 6.0–9.0 ppm, it would be expected to be rich in chemical information for important primary and secondary metabolites, such as the aromatic amino acids, tyrosine (δ_H_ 6.72 and 7.19 ppm), and tryptophan (δ_H_ 7.04 ppm), as well as for phenolic and/or polyphenolic substances, such as the isoflavones glycitin (δ_H_ 6.36 ppm), genistein (δ_H_ 6.68 ppm), and daidzein (δ_H_ 6.91 ppm), and metabolic products resulting from the shikimic acid pathway and directly influenced by the genetic modification of soy.^2,44,48^ However, the assignment of minor components was difficult because of the scarcity of data on these metabolic compounds in the literature.^34^ In the aromatic region, despite the low signal-to-noise ratio, some compounds were identified, such as fumaric acid (δ_H_ 6.65 ppm), gallic acid (δ_H_ 6.89 ppm), histidine (δ_H_ 7.97 ppm), and trigonelline (δ_H_ 8.86 ppm)^2^ confirmed from 2D NMR experiments.

**Figure 5:**
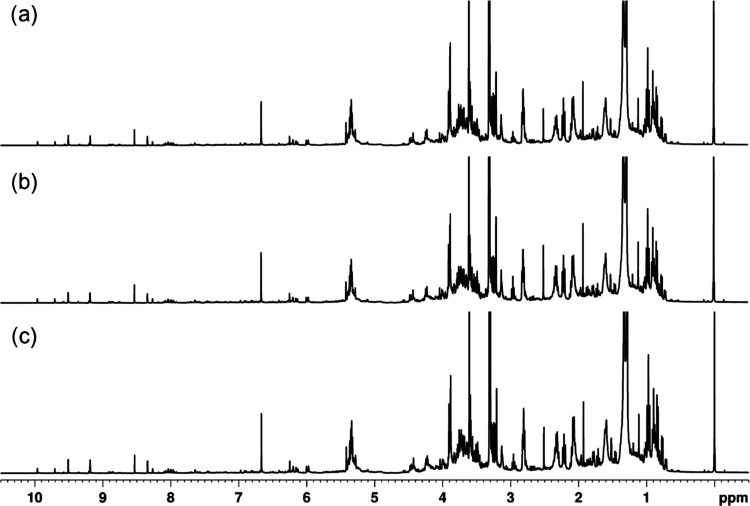
^1^H NMR spectra (400 MHz) of CD_3_OD extracts for Intact soybean leaves from groups: (a) T0 control group, (b) T1 AgNPs group, and (c) T2 AgNO_3_ group.

**Figure 6:**
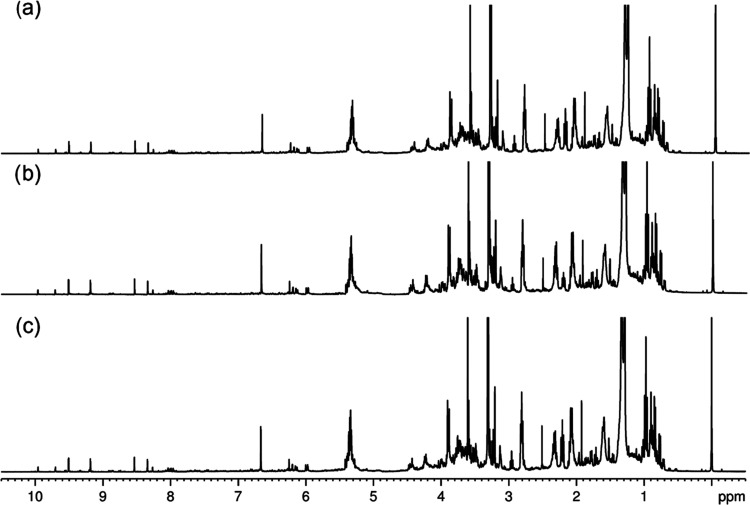
^1^H NMR spectra (400 MHz) of CD_3_OD extracts for RR soybean leaves from groups: (a) T0 control group, (b) T1 AgNPs group, and (**c**) T2 AgNO_3_ group.

When comparing the production of metabolites in the three treatments using the ^1^H NMR spectra of Intact soybean leaves and RR soybean leaves, little or no difference was observed in any of the comparisons. Regardless of genotype (Intact and RR plants) or treatment conditions, all ^1^H NMR spectra of extracts from the same tissue type (leaves or stem or roots) share the same signals, which may mean that they exhibit similar behaviors in the face of different treatments, although their relative intensity is variable. Figures 5 and 6 show the comparison of leaf spectra for the three treatments of Intact and RR soybean plants, respectively. Despite the low presence of phenolic compounds in the soybean metabolome in the methanolic extract of the three compartments, the region of δ_H_ 6.00–10.50 ppm was used in the exploratory PCA analysis of each one. The ^1^H spectra of the other compartments are shown in the Supporting Information (Figures S19–S22).

### 3.5 PCA Analysis

**Figure 7:**
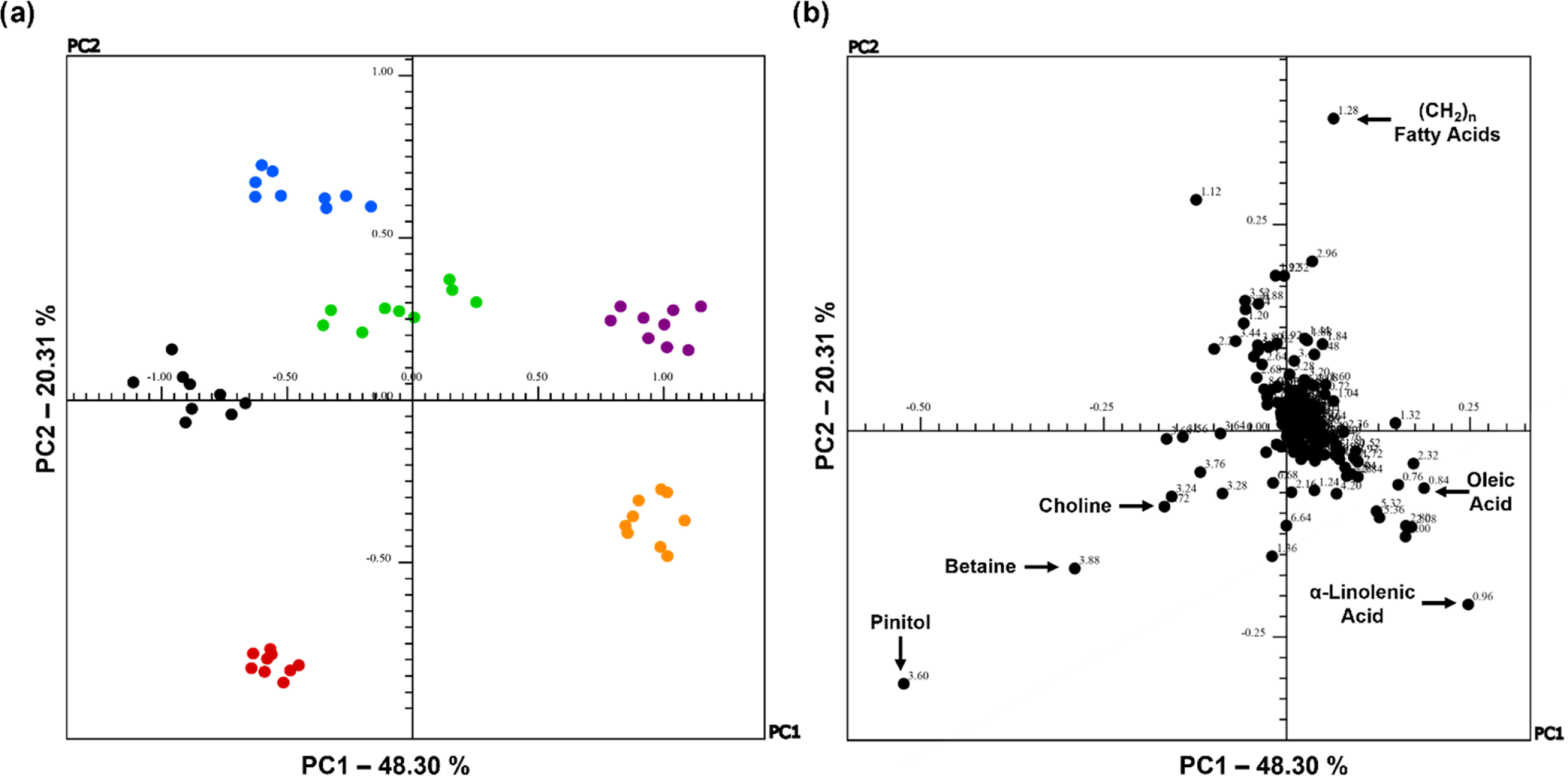
(a) Scores and (b) loadings graphs of PC1 versus PC2 for ^1^H NMR spectra of CD_3_OD extracts from leaves for discriminant analysis between treatments T0-I (black), T1-I (blue), T2-I (green), T0-RR (red), T1-RR (orange), and T2-RR (purple) of the transgenic soybean varieties Intact and RR.

**Figure 8:**
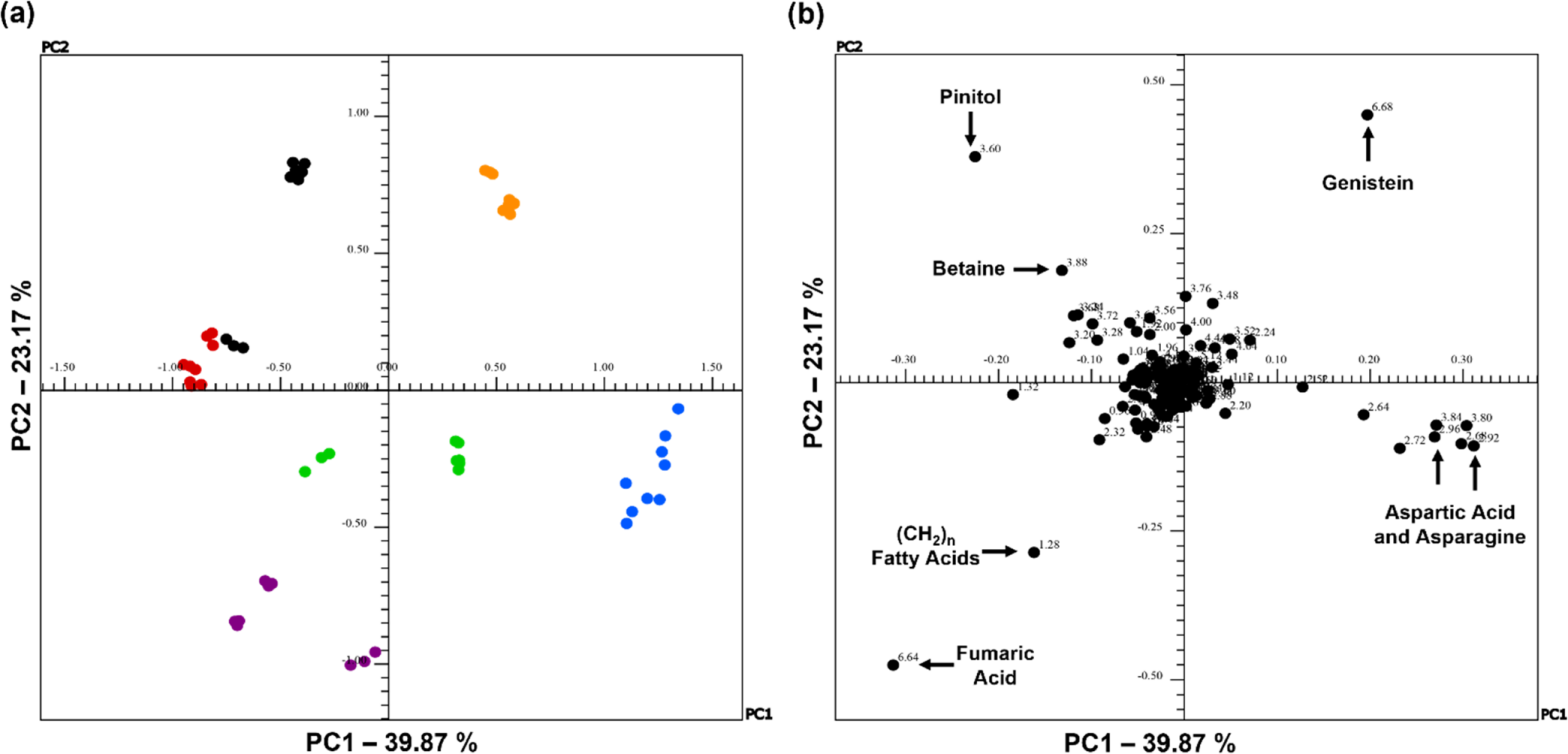
(a) Scores and (b) loadings graphs of PC1 versus PC2 for ^1^H NMR spectra of CD_3_OD extracts from stems for discriminant analysis between treatments T0-I (black), T1-I (blue), T2-I (green), T0-RR (red), T1-RR (orange), and T2-RR (purple) of the transgenic soybean varieties Intact and RR.

**Figure 9:**
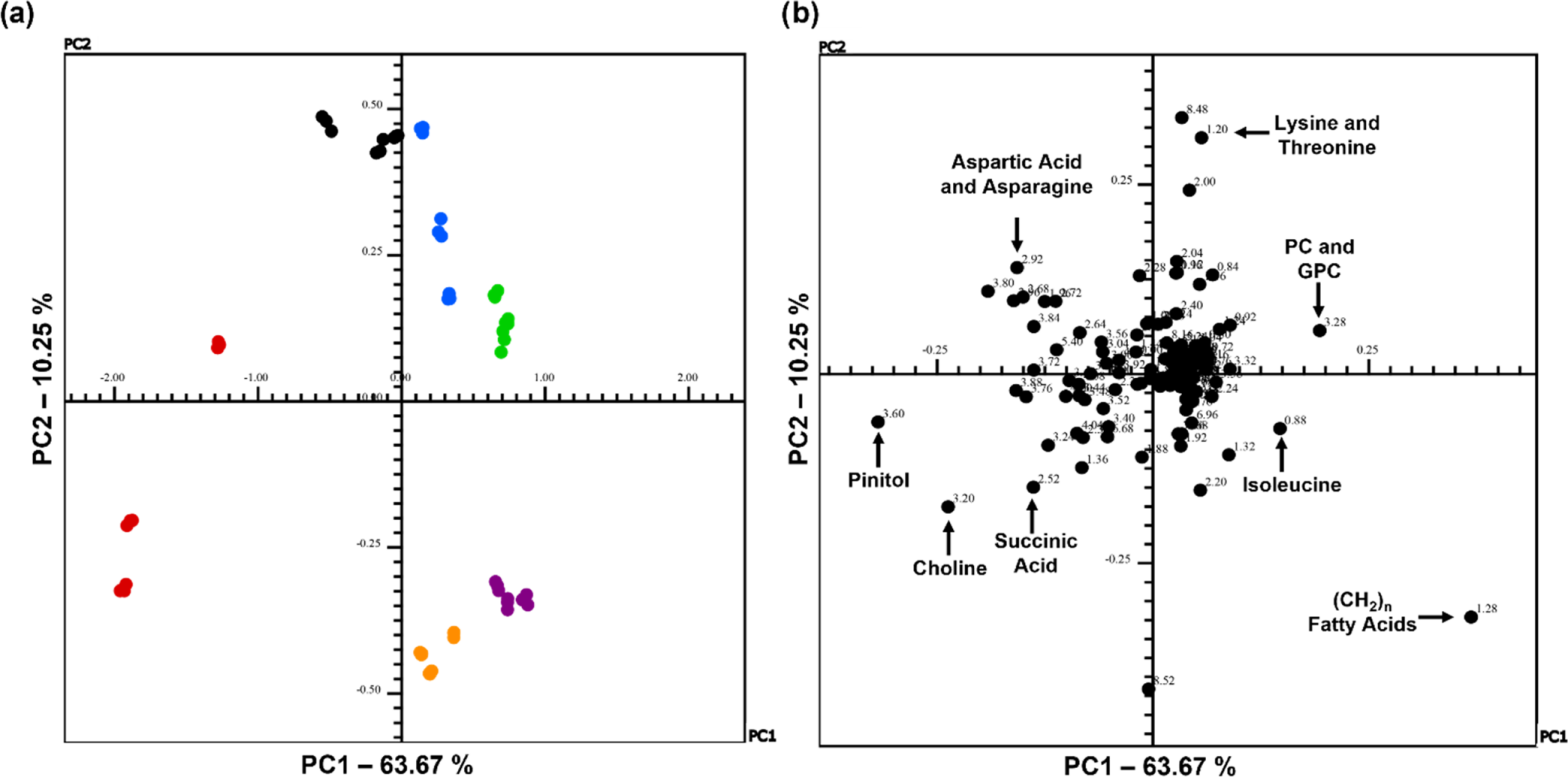
(a) Scores and (b) loadings graphs of PC1 versus PC2 for ^1^H NMR spectra of CD_3_OD extracts from roots for discriminant analysis between treatments T0-I (black), T1-I (blue), T2-I (green), T0-RR (red), T1-RR (orange) and T2-RR (purple) of the transgenic soybean varieties Intact and RR.

To compare the data set, unsupervised multivariate statistical analysis (principal component analysis, PCA) was used, allowing for discrimination of the samples according to the varieties, maintaining the maximum possible variability within the sample set, as well as identifying which regions of the ^1^H spectra are responsible for possible differences between samples. Figures 7–9 show the effect of preprocessing using Pareto scaling in discriminating the different compartments of *G. max* (L). Pareto scaling is very similar to autoscaling. However, the data is mean-centered and divided by the square root of the standard deviation. Thus, the more intense signals decrease more than the less intense signals in relation to the mean-centered data, thus reducing the relative importance of the intense signals. In the Pareto scale, the data structure is partially intact.^49^

The PCA score graph of the ^1^H NMR spectra of the leaf extracts is shown in Figure 7. A clear discrimination is observed between the leaves of the Intact groups and those of the RR group. This distinction occurred in the first two main components, which correspond to 68.61% of the explained variance (PC1 48.30% and PC2 20.31%). Considering all of the ^1^H NMR spectra derived from the leaves, the score graph of the first two PCs obtained shows a favorable separation of the six groups (T0-I, T1-I, T2-I, T0-RR, T1-RR, and T2-RR) along the two axes. It can be argued as a previous result that this separation is due to the type of transgenic variety (Intact vs RR) since the control and treated groups for the same variety remain together. Most Intact soybean samples are PC2 positive, while RR soybean samples are distributed in PC1 positive, except for those in the T0-RR group (control), which are PC1 negative. The loading graphs show that the metabolites of the saturated aliphatic chain, betaine (23), and pinitol (29) differentiate the leaves of the two soybean varieties (positive side of PC1). This behavior of separation data between samples of Intact leaves and RR leaves along the PC1 axis appears to be attributed to the buckets of saturated aliphatic chain compounds (δ_H_ 1.28 ppm), pinitol (δ_H_ 3.60 ppm), choline (δ_H_ 3. 72 ppm) (41), and betaine (δ_H_ 3.90 ppm) (23) for the Intact variety. For the leaves of the RR variety, the T1-RR and T2-RR groups are separated along negative PC2 due to the buckets referring to compounds as oleic acid (δ_H_ 0.84 ppm) (2) and α-linolenic acid (δ_H_ 0.97 ppm) (4) (Figure 7b).

The first two principal components of the stem extracts explained 39.87 and 23.17% of the total variance of the data when using Pareto scaling, respectively. The two control groups (T0-I and TRR) were discriminated from the other groups and grouped on the positive side of PC2, while the others were distributed throughout PC1. The groups that had AgNPs added, blue and orange, were on the positive side of PC1, which may suggest that the chemical composition was not so affected by the presence of the nanoparticle, and this corroborates the results of the electron micrograph analyses in which no significant changes were observed in the stem compartment when compared to the group treated with AgNO_3_. Another indication of this discrimination is that the three groups for both varieties were separated, proposing that the metabolites are affected differently for each treatment.

The differentiation between the groups is evident from the loadings graph (Figure 8b). Analysis of the graph allowed us to identify the metabolites responsible for the discrimination of the six groups. The T0-I and T0-RR groups were grouped into positive PC2 and differentiated from the others, mainly due to the presence of the bucket at δ_H_ 3.60 ppm attributed to pinitol (34), considered a chemical marker of *G. max* (L), which was confirmed in this work, and betaine (δ_H_ 3.90 ppm) (23) signals. It was noted that the T1-I and T1-RR groups were discriminated by the buckets referring to the aspartic acid (17), asparagine (18) (δ_H_ 2.92–2.96 ppm), and genistein (δ_H_ 6.68 ppm) (35), on the positive axis of PC1. Furthermore, the differentiation of the T2-I and T2-RR groups in relation to the other groups occurred mainly in the buckets referring to the signals of the saturated aliphatic chain (δ_H_ 1.28 ppm) and fumaric acid (δ_H_ 6.65 ppm) (30).

The first two main components of the PCA of the root extracts obtained with the Pareto scale explained 73.92% of the total variance of the data (Figure 9a,b). It is noteworthy that the groups for each treatment were separated practically, one in each quadrant. A clear separation was observed between the roots of the control groups, which were on the side of negative PC1 and positive PC2, with a bucket of δ_H_ 2.92 ppm, referring to the aspartic acid (17) and asparagine (18), amino acids responsible for grouping these samples. The results also showed the presence of succinic acid (δ_H_ 2.50 ppm) (28), pinitol (δ_H_ 3.60 ppm) (29), and choline (δ_H_ 3.20 ppm), which were responsible for grouping the samples from the RR-T0 group, from the groups treated with AgNPs and AgNO_3_. The samples from the T2 group for both varieties, purple and green, were grouped in the two PC2 axes, T2-I in positive PC2 and T2-RR in negative PC2, showing that satisfactory discrimination is occurring between the two types of soybeans studied. For samples T1-I and T2-I, they were grouped in the positive region of PC1 and PC2, making it possible to state that the distinction between the groups occurs mainly due to the presence of buckets of δ_H_ 1.20 ppm and δ_H_ 3.28 ppm attributed to lysine (10) and threonine (11), and phosphoricoline (42) and glycerophosphoricoline (43), respectively. The metabolites responsible for grouping the samples from groups T1-RR and T2-RR, grouped in the positive region of PC1 and negative region of PC2, were distributed in the buckets associated with isoleucine (δ_H_ 0.88 ppm) (8) and saturated aliphatic chain compounds (δ_H_ 1.28 ppm).

The scores graph of the soybean roots showed better discrimination between the different treatments than the scores graph of the leaf and stem extracts, Figures 7 and 8, respectively. The results observed in the identification of metabolites and PCA corroborate the characteristics of the soybean species studied, confirming that NMR-based metabolomics offers consistent results. However, PCA did not reveal so many significant phenotypic differences between the two varieties studied since the compounds responsible for separating the groups are recurrent in leaves, stems, and roots.

In summary, this study has shown that nuclear magnetic resonance spectroscopy and chemometrics can be used to differentiate varieties of transgenic soybean plants at their V3 stage of development in three different treatments. From the analysis of the ^1^H NMR spectra of the three compartments, it was possible to identify the presence of fatty acids, amino acids, organic acids, sugars, and polyphenols and the presence of some isoflavones considered biomarkers for soybeans. According to the microscopy results, Ag absorption in roots and leaves of soybean plants was greater after exposure to AgNO_3_ treatment for both varieties, which may be correlated with the oxidative stress parameters caused by Ag in ionic form. The root was the most affected compartment for both treatments, in which the presence of cracks was diagnosed, similar to those that occurred in the stem region. The growth and quality of plants in the V3 stage are not affected by exposure to AgNPs, showing that there is no toxicity of AgNPs for plants grown in soil. Our results may lead to a better understanding of the presence of NPs in soybean crops, suggesting that silver in the form of ions affects the growth of plants more than nanoparticle silver. This indicates that plants may be more sensitive to treatment with ionic silver, as the appearance of chlorosis was observed in the leaves of the two transgenic varieties. The discriminant analysis of main components for the leaves showed that there is a difference between the RR variety and the Intact and pointed out that fatty acids, betaine, choline, and pinitol are the markers that differentiate the two varieties. Therefore, the results demonstrated so far reflect the importance of studying the effects that AgNPs have on soybean crops, and correlating which compounds are responsible for these changes is relevant for research. Furthermore, additional studies, such as the morphology and anatomy of plants involving NPs and their interaction with organisms, are always welcome to expand information on the possible risks of their use in agricultural products derived from the use or contamination with nanoparticles.

## Supporting Information Available

Scanning electron micrographs in the leaf compartment for RR and Intact soybean plants (Figure S1); scanning electron micrographs (a–f) and light photomicrographs (g–o) were performed to analyze the characteristics of the stem surfaces (Figure S2); scanning electron micrographs (a–c) and light photomicrographs (d–i) were performed to analyze the characteristics of the root surfaces (PDF)

## Abbreviations Used

Ala: alanine
Arg: arginine
Cho: choline
FA: fatty acids
Fru: fructose
GABA: 4-aminobutyrate
Glc: glucose
Gln: glutamine
Glu: glutamic acid
Gly: glycine
GPC: glycerophosphorylcholine
His: histidine
Isoleu: isoleucine
Leu: leucine
Lys: lysine
NI: nonidentified
Trp: tryptophan
Thr: threonine
Trig: trigonelline
Tyr: tyrosine
Val: valine
