# Supplementary material for: Influence of Silver Nanoparticles on the Metabolites of Two Transgenic Soybean Varieties: An NMR-Based Metabolomics Approach
Source: J Agric Food Chem. 2024 May 15;72(21):12281–94. doi: 10.1021/acs.jafc.4c00756 (PMC11140748; doi:10.1021/acs.jafc.4c00756)
Supplement: Supplementary file 1 — jf4c00756_si_001.pdf [file jf4c00756_si_001.pdf]

Supporting Information

Influence of Silver Nanoparticles on The Metabolites of Two Transgenic Soybean Varieties: an NMR-Based Metabolomics Approach

Amanda L. Quintela[a,d], Maria F. C. Santos[a], Rodrigo F. de Lima[b], Juliana L. S. Mayer[b], Gustavo G. Marcheafave[c], Marco A. Z. Arruda[d] and Cláudio F. Tormena[a*]

*e-mail: tormena@unicamp.br

[a] Physical Organic Chemistry Laboratory, Institute of Chemistry, Universidade Estadual de Campinas, UNICAMP, PO Box 6154, Campinas, São Paulo 13083-970, Brazil.

[b] Laboratory of Plant Anatomy, Institute of Biology, Universidade Estadual de Campinas, UNICAMP, PO Box 6109, Campinas, São Paulo 13083-862, Brazil.

[c] Institute of Chemistry, Universidade Estadual de Campinas, UNICAMP, PO Box 6154, Campinas, São Paulo 13083-970, Brazil.

[d] Spectrometry, Sample Preparation and Mechanization Group, Institute of Chemistry, Universidade Estadual de Campinas, UNICAMP, PO Box 6154, Campinas, São Paulo 13083-970, Brazil.

Contents

1.1 Microscopy 5
Figure S1. Scanning electron micrographs (**a, b, c, d, e, f, i, l**) and light photomicrographs (**g, h, j, k, m, n, o**) were used to analyze the characteristics of the leaves. In conventional (**a**), RR (**b**) and Intact (**c**) varieties, the juxtaposition of epidermal cells is observed with the presence of non-glandular trichomes and deposition of epicuticular waxes. In the scanning electron micrograph of leaves treated with AgNPs (**d**), it is possible to visualize the remaining base of the trichome. On the abaxial and adaxial surfaces of the leaves treated with $AgNO_3$ (**e, f**), the presence of veins with cracks can be noted, seen in more detail in (**l** - arrow), as well as trichome gaps, highlighted in figure (**i**). In relation to the cross sections of the midrib in the different treatments (control, $AgNO_3$ and AgNPs) (**g, j, h, k**), degradation of parenchyma cells in the abaxial region of the midrib is observed, indicated by arrows (h), not present in the control (**g, j**) and AgNPs (**k**) treatments. In the cross-sections of the leaf mesophyll in the control (**m**), AgNPs (**m**) and $AgNO_3$ (**n, o**) treatments, it is possible to observe the curling and/or detachment of the abaxial epidermis, also indicated by arrows. Bars: 500μm = a, b, c, d, e, f. 50μm = g, h, i, j, k, l, m, n, o. Ep = epidermis; Nt = non-glandular trichome; Gt = glandular trichome; Vb = vascular bundle; L = trichome gap; Pp = palisade parenchyma; Lp = lacune parenchyma; St = stoma. The bars indicate the amplification as follows: 4x = 500μm, 10x = 200μm, 20x = 100μm, 40x = 50μm, 100x = 20μm. All cross-sections are stained with Toluidine Blue pH 4.7..........6
Figure S2. Scanning electron micrographs (**a, b, c, d, e, f**) and light photomicrographs (**g - o**) were performed to analyze the characteristics of the stem surfaces. On the stem surface of the control treatment of the RR (**a**) and Intact (**b**) varieties, the presence of glandular and non-glandular trichomes was observed. In the treatment with AgNPs, there is a decrease in the number of trichomes and the beginning of small cracks (**c**). In plants treated with $AgNO_3$, there is implosion of epidermal cells (**d**), cracks (**e**) and detachment of the epidermis (**f**). In longitudinal sections of the stem region in the control treatment, epidermal and cortical parenchymatic cells were observed without damage and without accumulation of intracellular material (**g - i**). In plants treated with AgNPs, there are cortical parenchyma cells in the beginning of degradation (**k**, *) with accumulation of phenolic compounds in the affected regions (**l**). In plants treated with $AgNO_3$, the implosion of epidermal cells (**m**, *), the accumulation of phenolic compounds (**n**) and the loosening of anticlinal cell walls (**o**), indicated by arrows, are observed. Bars: 500 μm= a, b, c. 250μm= d. 200μm= g, j, m. 100μm= e, f, n. 50μm = h, i, k, l, o. Ep = epidermis; Nt = non-glandular trichome; Gt = glandular trichome; Cp = cortical parenchyma; Vb = vascular bundle; Pit = marrow. The bars indicate the amplification as follows: 4x = 500μm, 10x = 200μm, 20x = 100μm, 40x = 50μm, 100x = 20μm. All cross-sections are stained with Toluidine Blue pH 4.7 7
Figure S3. Scanning electron micrographs (**a, b, c**) and light photomicrographs (**d - i**) were performed to analyze the characteristics of the root surfaces. In the control treatment, the root surface (**a**) shows intact epidermal cells. In plants treated with AgNPs, the beginning of epidermis detachment and small cracks were observed (**b**). In plants treated with $AgNO_3$, it is common to find degraded epidermal cells and cracks on the root surfaces (**c**). In the transverse and longitudinal sections of the roots in the control treatment (**d, g, j**), the integrity of the epidermal and parenchymatic cells can be seen. In roots treated with AgNPs, the beginning of epidermal cell implosion, loosening of cell walls and irregularity in intercellular spaces (**e, h, k**) were observed. In roots treated with $AgNO_3$, the implosion of epidermal cells (**f**, *), loosening of the cell walls of the epidermis and cortical parenchyma, as well as irregular intercellular spaces (**i**) stand out. In (**l**), degraded epidermal cells are demonstrated, indicated by the arrow, and the accumulation of phenolic compounds in affected areas. Bars: 250μm = a, b, c. 200μm = d, e, f. 50μm = i, g, h, l. 20μm = k. Ep = epidermis; Lr = lateral root; Cp = cortical parenchyma; CV = vascular cylinder; Pit = marrow; Is = intercellular space. The bars indicate the amplification as follows: 4x = 500μm, 10x = 200μm, 20x = 100μm, 40x = 50μm, 100x = 20μm. 8
Figure S4. Autofluorescence photomicrographs (**a – e**) and after reaction with Calcofluor White (**f – i**). In the root cross section under autofluorescence filter in the control treatment, the integrity of the cells is observed (**a**). In plants treated with AgNPs, it is possible to observe the beginning of the accumulation of phenolic compounds in the parenchyma just below the epidermis, as well as irregular epidermal cells (**b**). In $AgNO_3$ treatment, cell irregularity is seen in the epidermis and throughout the root cortex, with accumulation of phenolic compounds and wide intercellular spaces (**c**). The longitudinal root (**d**) and stem (**e**) sections show the presence of phenolic compounds in regions already affected and in regions at the beginning of the degradation process. In root cross-sections after the reaction with Calcofluor, it is possible to observe the presence of cellulose in the cell wall of epidermal, cortical and vascular cylinder cells in the control treatment (**f**). In treatments with $AgNO_3$ and AgNPs, the biopolymer is not visible in the affected regions, concomitant with the accumulation of phenolic compounds (**g, h, i**). Bars: 100 μm = i. 50 μm = a, b, c, d, e, f, g, h. Ep =

epidermis; Lr = lateral root; Cp = cortical parenchyma; CV = vascular cylinder; Ph = phenolic compounds; Is = intercellular space. The bars indicate the amplification as follows: 4x = 500μm, 10x = 200μm, 20x = 100μm, 40x = 50μm, 100x = 20μm. 9

1.2 .... Identification and comparison of metabolite profiles present in transgenic soybean plants. 10

Figure S5. Representation of the 2D NMR spectrum of (^{1}H-^{1}H) COSY (600 MHz) from CD_3OD extracts for soybean leaves from the control group. 10

Figure S6. Representation of the 2D NMR spectrum of multiplicity-edited (^{1}H-^{13}C)HSQC ^{1}H-^{13}C (600 MHz) from CD_3OD extracts for soybean leaves from the control group. 11

Figure S7. Representation of the 2D NMR spectrum of HSQC-TOCSY (600 MHz) from CD_3OD extracts for soybean leaves from the control group. 11

Figure S8. Representation of the 2D NMR spectrum of HMBC ^{1}H-^{13}C (600 MHz) from CD_3OD extracts for soybean leaves from the control group. 12

Figure S9. Representation of the 2D NMR spectrum of COSY ^{1}H-^{1}H (600 MHz) from CD_3OD extracts for soybean stem from the control group. 12

Figure S10. Representation of the 2D NMR spectrum of multiplicity-edited HSQC ^{1}H-^{13}C (600 MHz) from CD_3OD extracts for soybean stem from the control group. 13

Figure S11. Representation of the 2D NMR spectrum of HSQC-TOCSY (600 MHz) from CD_3OD extracts for soybean stem from the control group. 13

Figure S12. Representation of the 2D NMR spectrum of HMBC ^{1}H-^{13}C (600 MHz) from CD_3OD extracts for soybean stem from the control group. 14

Figure S13. Representation of the 2D NMR spectrum of selective HMBC ^{1}H-^{13}C (600 MHz) from CD_3OD extracts for soybean stem from the control group. 14

Figure S14. Representation of the 2D NMR spectrum of COSY ^{1}H-^{1}H (600 MHz) from CD_3OD extracts for soybean root from the control group. 15

Figure S15. Representation of the 2D NMR spectrum of multiplicity-edited HSQC ^{1}H-^{13}C (600 MHz) from CD_3OD extracts for soybean root from the control group. 15

Figure S16. Representation of the 2D NMR spectrum of HSQC-TOCSY (600 MHz) from CD_3OD extracts for soybean root from the control group. 16

Figure S17. Representation of the 2D NMR spectrum of selective HMBC ^{1}H-^{13}C (600 MHz) from CD_3OD extracts for soybean root from the control group. 16

Figure S18. Chemical structures of assigned compounds. 18

Figure S19. Representative ^{1}H NMR spectra (400 MHz) with suppression of the residual water signal of CD_3OD extracts for Intact soybean stem from groups (**a**) T0 control group, (**b**) T1 AgNPs group and (**c**) T2 $AgNO_3$ group. 19

Figure S20. Representative ^{1}H NMR spectra (400 MHz) with suppression of the residual water signal of CD_3OD extracts for RR soybean stem from groups (**a**) T0 control group, (**b**) T1 AgNPs group and (**c**) T2 $AgNO_3$ group. 19

Figure S21. Representative ^{1}H NMR spectra (400 MHz) with suppression of the residual water signal of CD_3OD extracts for Intact soybean root from groups (**a**) T0 control group, (**b**) T1 AgNPs group and (**c**) T2 $AgNO_3$ group. 20

Figure S22. Representative ^{1}H NMR spectra (400 MHz) with suppression of the residual water signal of CD_3OD extracts for RR soybean root from groups (**a**) T0 control group, (**b**) T1 AgNPs group and (**c**) T2 $AgNO_3$ group. 20

1.1 Microscopy

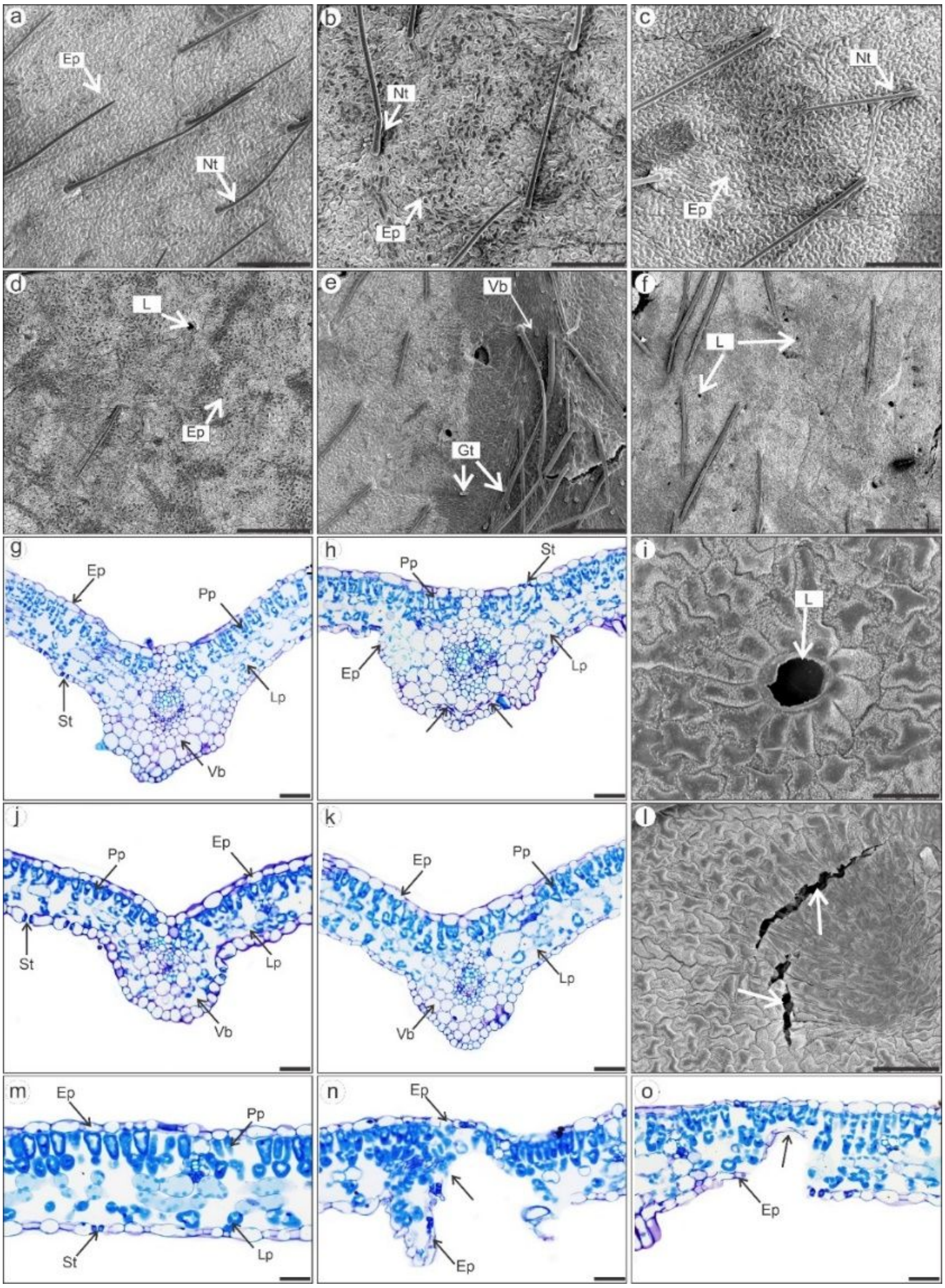

Figure S1. Scanning electron micrographs (**a, b, c, d, e, f, i, l**) and light photomicrographs (**g, h, j, k, m, n, o**) were used to analyze the characteristics of the leaves. In conventional (**a**), RR (**b**) and Intact (**c**) varieties, the juxtaposition of epidermal cells is observed with the presence of non-glandular trichomes and deposition of epicuticular waxes. In the scanning electron micrograph of leaves treated with AgNPs (**d**), it is possible to visualize the remaining base of the trichome. On the abaxial and adaxial surfaces of the leaves treated with $AgNO_3$ (**e, f**), the presence of veins with cracks can be noted, seen in more detail in (**l** - arrow), as well as trichome gaps, highlighted in figure (**i**). In relation to the cross sections of the midrib in the different treatments (control, $AgNO_3$ and AgNPs) (**g, j, h, k**), degradation of parenchyma cells in the abaxial region of the midrib is observed, indicated by arrows (h), not present in the control (**g, j**) and AgNPs (**k**) treatments. In the cross-sections of the leaf mesophyll in the control (**m**), AgNPs (**m**) and $AgNO_3$ (**n, o**) treatments, it is possible to observe the curling and/or detachment of the abaxial epidermis, also indicated by arrows. Bars: 500µm = a, b, c, d, e, f. 50µm = g, h, i, j, k, l, m, n, o. Ep = epidermis; Nt = non-glandular trichome; Gt = glandular trichome; Vb = vascular bundle; L = trichome gap; Pp = palisade parenchyma; Lp = lacune parenchyma; St = stoma. The bars indicate the amplification as follows: 4x = 500µm, 10x = 200µm, 20x = 100µm, 40x = 50µm, 100x = 20µm. All cross-sections are stained with Toluidine Blue pH 4.7

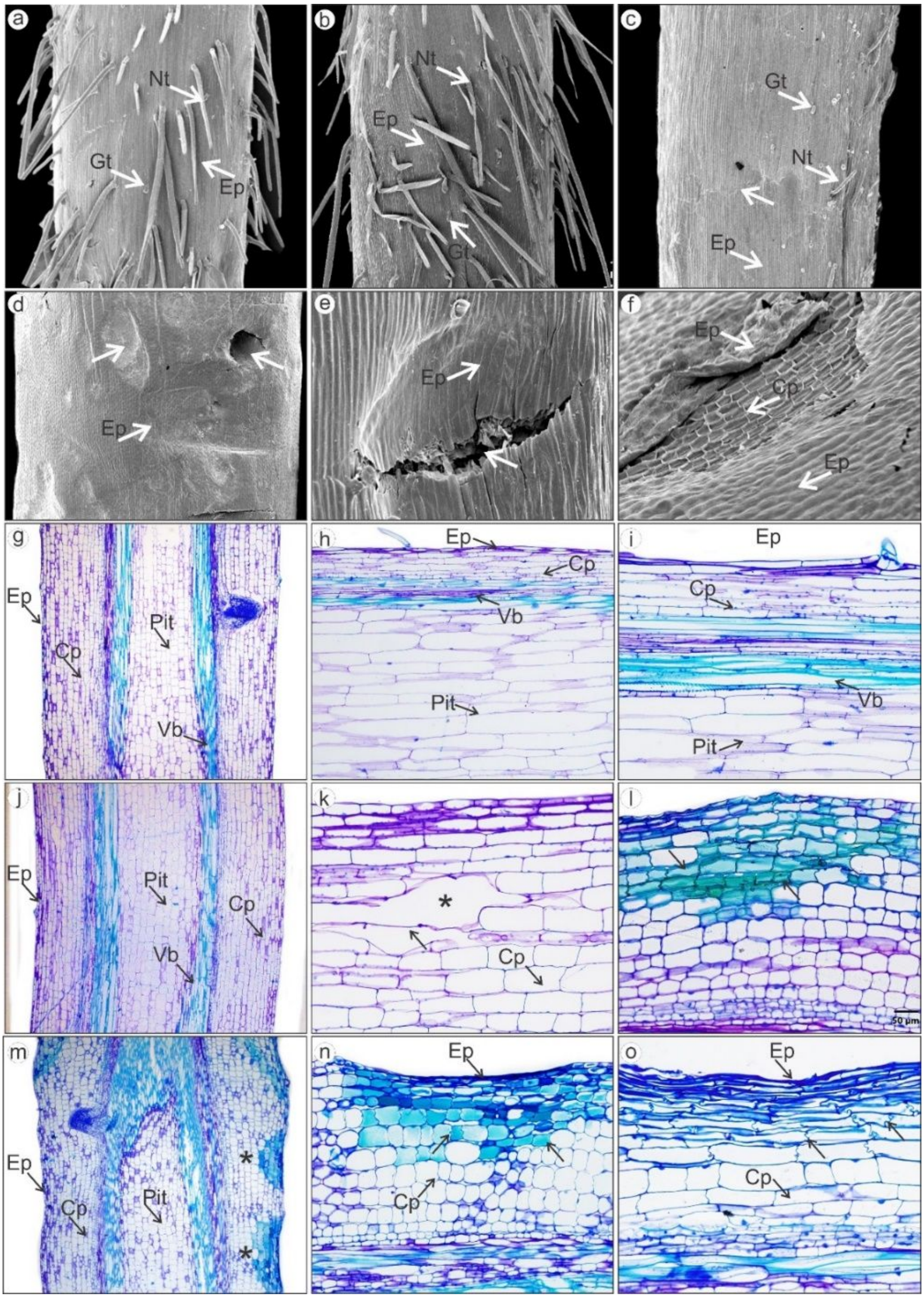

Figure S2. Scanning electron micrographs (**a, b, c, d, e, f**) and light photomicrographs (**g - o**) were performed to analyze the characteristics of the stem surfaces. On the stem surface of the control treatment of the RR (**a**) and Intact (**b**) varieties, the presence of glandular and non-glandular trichomes was observed. In the treatment with AgNPs, there is a decrease in the number of trichomes and the beginning of small cracks (**c**). In plants treated with $AgNO_3$, there is implosion of epidermal cells (**d**), cracks (**e**) and detachment of the epidermis (**f**). In longitudinal sections of the stem region in the control treatment, epidermal and cortical parenchymatic cells were observed without damage and without accumulation of intracellular material (**g - i**). In plants treated with AgNPs, there are cortical parenchyma cells in the beginning of degradation (**k**, *) with accumulation of phenolic compounds in the affected regions (**l**). In plants treated with $AgNO_3$, the implosion of epidermal cells (**m**, *), the accumulation of phenolic compounds (**n**) and the loosening of anticlinal cell walls (**o**), indicated by arrows, are observed. Bars: 500 µm= a, b, c. 250µm= d. 200µm= g, j, m. 100µm= e, f, n. 50µm = h, i, k, l, o. Ep = epidermis; Nt = non-glandular trichome; Gt = glandular trichome; Cp = cortical parenchyma; Vb =

vascular bundle; Pit = marrow. The bars indicate the amplification as follows: 4x = 500µm, 10x = 200µm, 20x = 100µm, 40x = 50µm, 100x = 20µm. All cross-sections are stained with Toluidine Blue pH 4.7

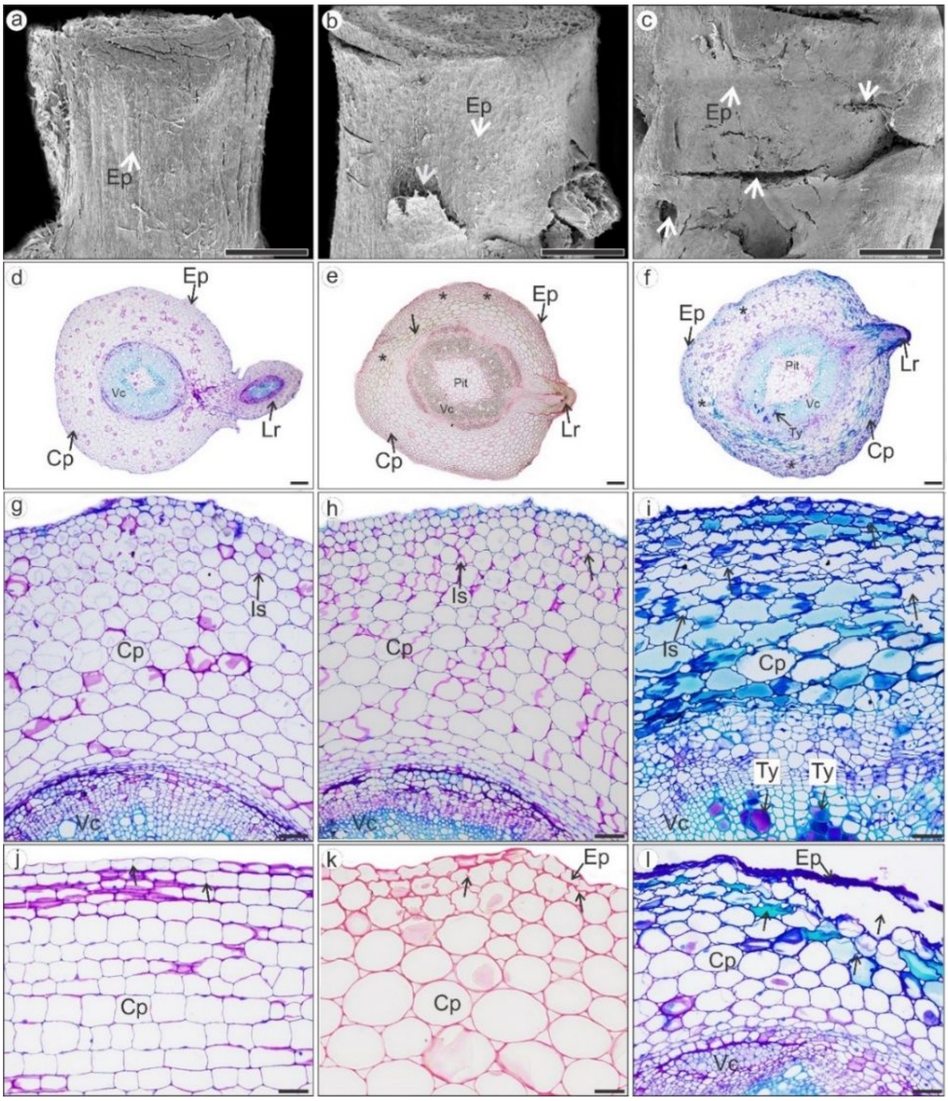

Figure S3. Scanning electron micrographs (**a, b, c**) and light photomicrographs (**d - i**) were performed to analyze the characteristics of the root surfaces. In the control treatment, the root surface (**a**) shows intact epidermal cells. In plants treated with AgNPs, the beginning of epidermis detachment and small cracks were observed (**b**). In plants treated with $AgNO_3$, it is common to find degraded epidermal cells and cracks on the root surfaces (**c**). In the transverse and longitudinal sections of the roots in the control treatment (**d, g, j**), the integrity of the epidermal and parenchymatic cells can be seen. In roots treated with AgNPs, the beginning of epidermal cell implosion, loosening of cell walls and irregularity in intercellular spaces (**e, h, k**) were observed. In roots treated with $AgNO_3$, the implosion of epidermal cells (**f**, *), loosening of the cell walls of the epidermis and cortical parenchyma, as well as irregular intercellular spaces (**i**) stand out. In (**l**), degraded epidermal cells are demonstrated, indicated by the arrow, and the accumulation of phenolic compounds in affected areas. Bars: 250µm = a, b, c. 200µm = d, e, f. 50µm = i, g, h, l. 20µm = k. Ep = epidermis; Lr = lateral root; Cp = cortical parenchyma; CV = vascular cylinder; Pit = marrow; Is = intercellular space. The bars indicate the amplification as follows: 4x = 500µm, 10x = 200µm, 20x = 100µm, 40x = 50µm, 100x = 20µm.

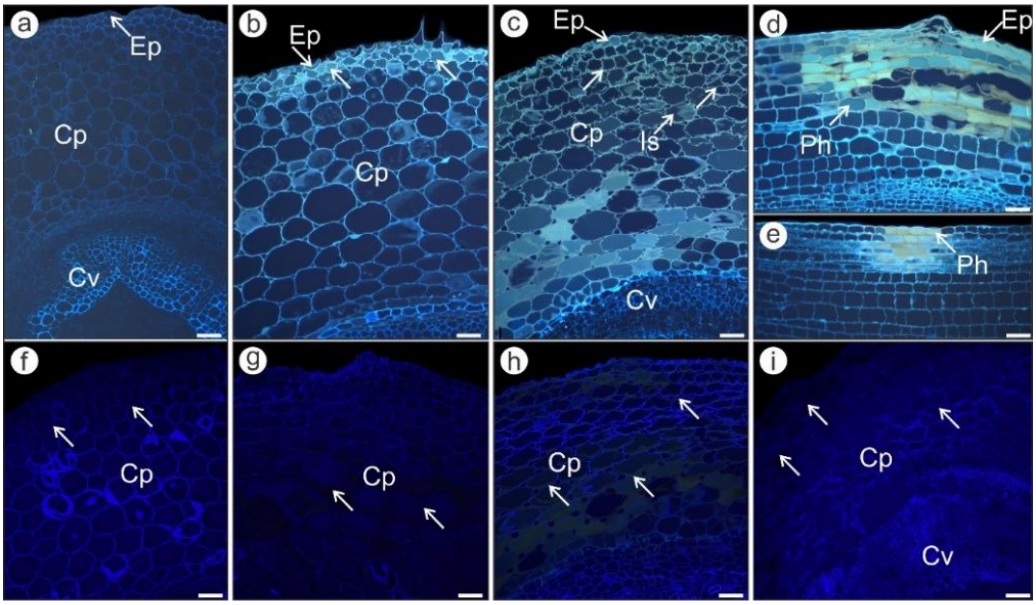

Figure S4. Autofluorescence photomicrographs (**a – e**) and after reaction with Calcofluor White (**f – i**). In the root cross section under autofluorescence filter in the control treatment, the integrity of the cells is observed (**a**). In plants treated with AgNPs, it is possible to observe the beginning of the accumulation of phenolic compounds in the parenchyma just below the epidermis, as well as irregular epidermal cells (**b**). In $AgNO_3$ treatment, cell irregularity is seen in the epidermis and throughout the root cortex, with accumulation of phenolic compounds and wide intercellular spaces (**c**). The longitudinal root (**d**) and stem (**e**) sections show the presence of phenolic compounds in regions already affected and in regions at the beginning of the degradation process. In root cross-sections after the reaction with Calcofluor, it is possible to observe the presence of cellulose in the cell wall of epidermal, cortical and vascular cylinder cells in the control treatment (**f**). In treatments with $AgNO_3$ and AgNPs, the biopolymer is not visible in the affected regions, concomitant with the accumulation of phenolic compounds (**g, h, i**). Bars: 100 µm = i. 50 µm = a, b, c, d, e, f, g, h. Ep = epidermis; Lr = lateral root; Cp = cortical parenchyma; CV = vascular cylinder; Ph = phenolic compounds; Is = intercellular space. The bars indicate the amplification as follows: 4x = 500µm, 10x = 200µm, 20x = 100µm, 40x = 50µm, 100x = 20µm.

1.2 Identification and comparison of metabolite profiles of the transgenic soybean plants.

The homo- and hetero-nuclear two-dimensional NMR experiments: COSY ^{1}H -^{1}H, HSQC ^{1}H-^{13}C, HSQC-TOCSY ^{1}H-^{13}C, and HMBC ^{1}H-^{13}C, were carried out to assist in the identification of the compounds. The ^{1}H-^{1}H homonuclear correlations of soybean leaf samples in CD_3OD were obtained by employing a COSY experiment with suppression of the residual OH signal from solvent, using the *cosygpprqf* pulse sequence. The following parameters were used in the acquisition: TD F2 of 2k TD F1 of 256, NS of 32 scans, SW at F2 and F1 of 12 ppm. The HSQC experiment with ^{1}H-^{13}C multiplicity editing were acquired with ^{13}C decoupling during acquisition, using the sequence *hsqcedetgpsisp* with TD F2 of 2k, TD F1 of 512, NS of 32, SW of 20 and 240 ppm in F2 and F1, respectively, and correlation time (D4) of 1.7 ms. The experiment for the observation of long-range ^{1}H-^{13}C heteronuclear correlations was obtained through HMBC with suppression of the residual OH signal from solvent, using the pulse sequence *hmbcgplpndprqf* with TD in F2 of 2k, TD F1 of 256, NS of 64 scans, SW of 20 and 211 ppm in F2 and F1, respectively, adjusting the evolution time (D6) to 62.5 ms. The selective HMBC experiment was also performed, using the shmbcctetgpl2nd pulse sequence with TD at F2 of 2k, TD F1 of 128, NS of 128 scans, SW of 20 and 7.71 ppm at F2 and F1, respectively, adjusting the transmitter frequency offset of carbon to 173 ppm and hydrogen to 4.87 ppm. To identify which carbonyls, correlate with the protons at 2.68 and 2.94, for the stem extract sample. The HSQC-TOCSY spectrum had suppression of the OH signal from solvent, using the *hsqcdiedetgpsisp* pulse sequence with TD F2 of 2k, TD F1 of 256, with a SW of 18 and 211 ppm at F2 and F1, respectively, correlation time (D4) of 1.7 ms and a mixing time (D9) of 60 ms. These same experiments were also carried out for stem and root extracts.

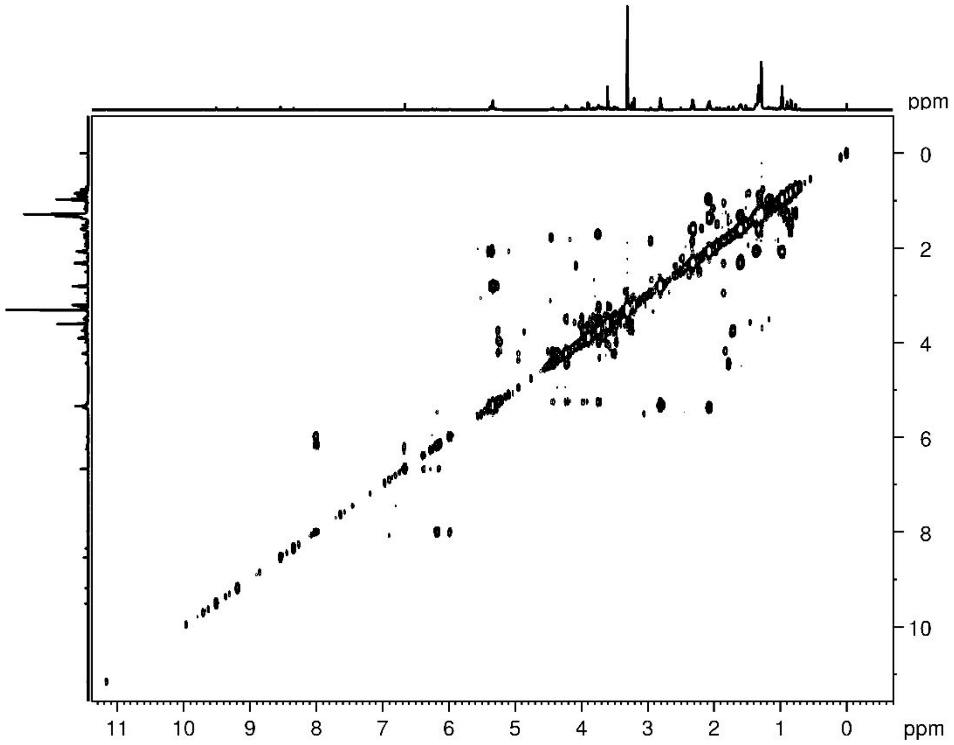

Figure S5. 2D NMR spectrum of COSY ^{1}H-^{1}H (600 MHz) from CD_3OD extracts for soybean leaves from the control group.

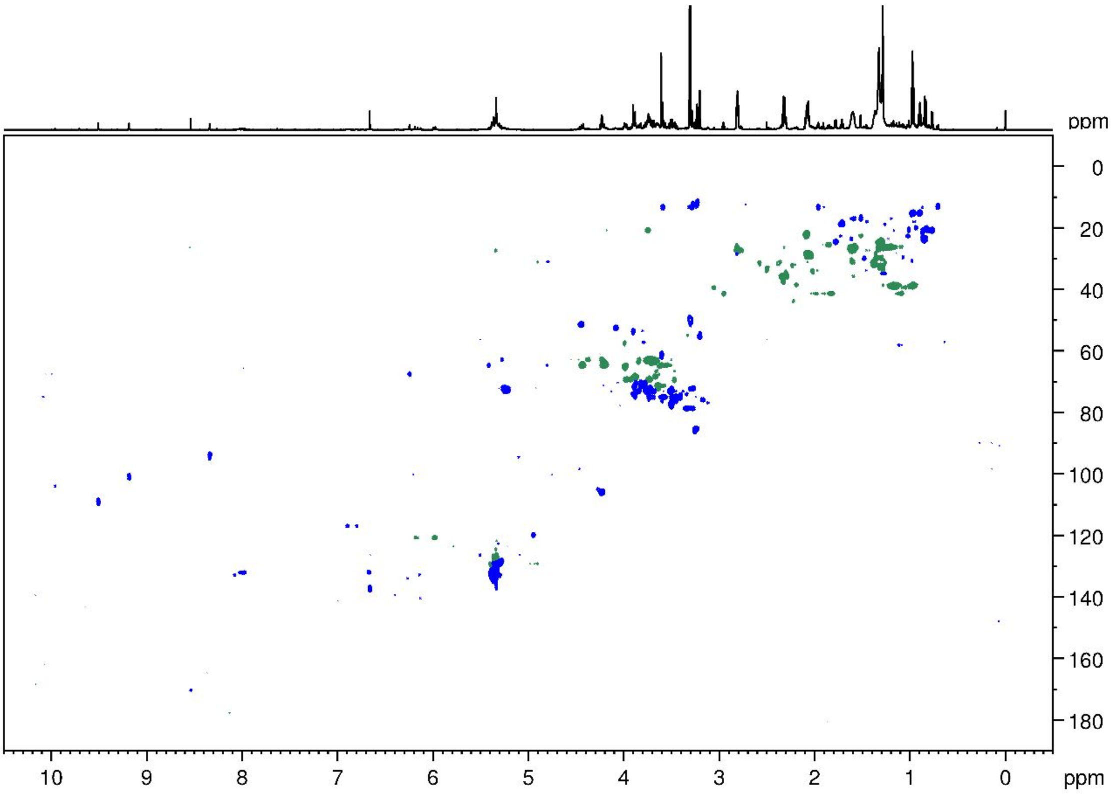

Figure S6. 2D NMR spectrum of multiplicity-edited HSQC ^{1}H-^{13}C (600 MHz) from CD_3OD extracts for soybean leaves from the control group.

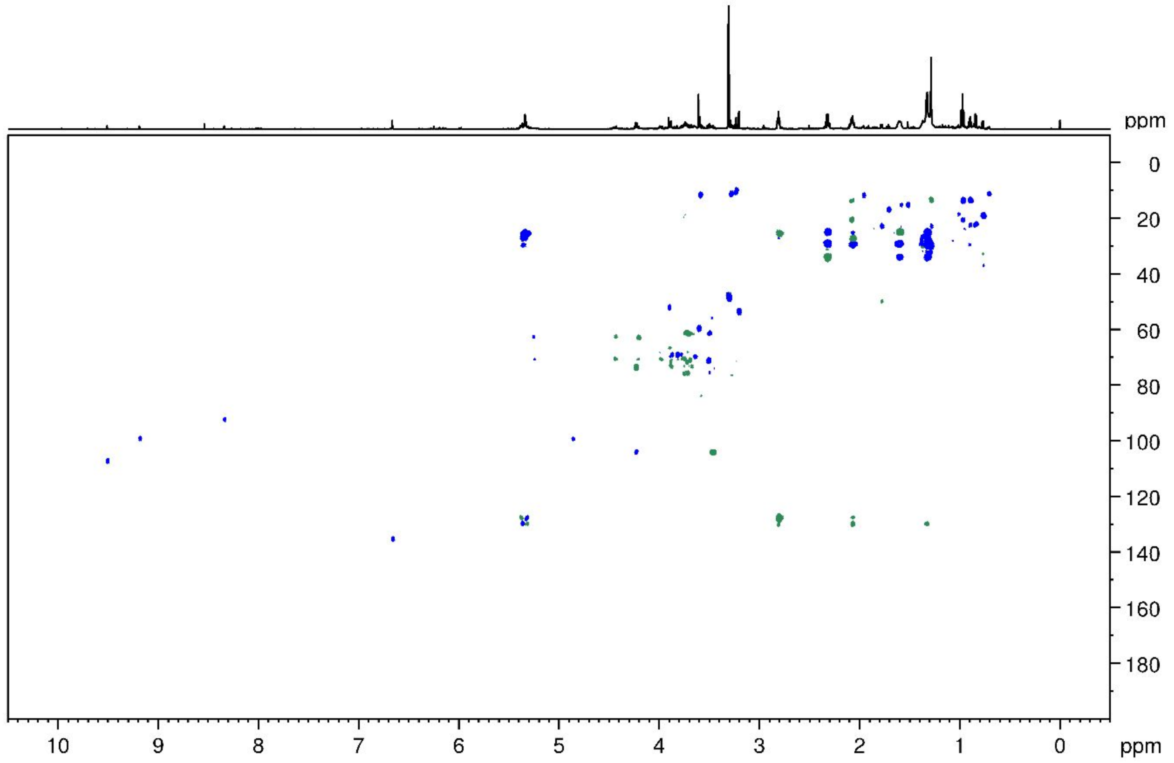

Figure S7. 2D NMR spectrum of HSQC-TOCSY (600 MHz) from CD_3OD extracts for soybean leaves from the control group.

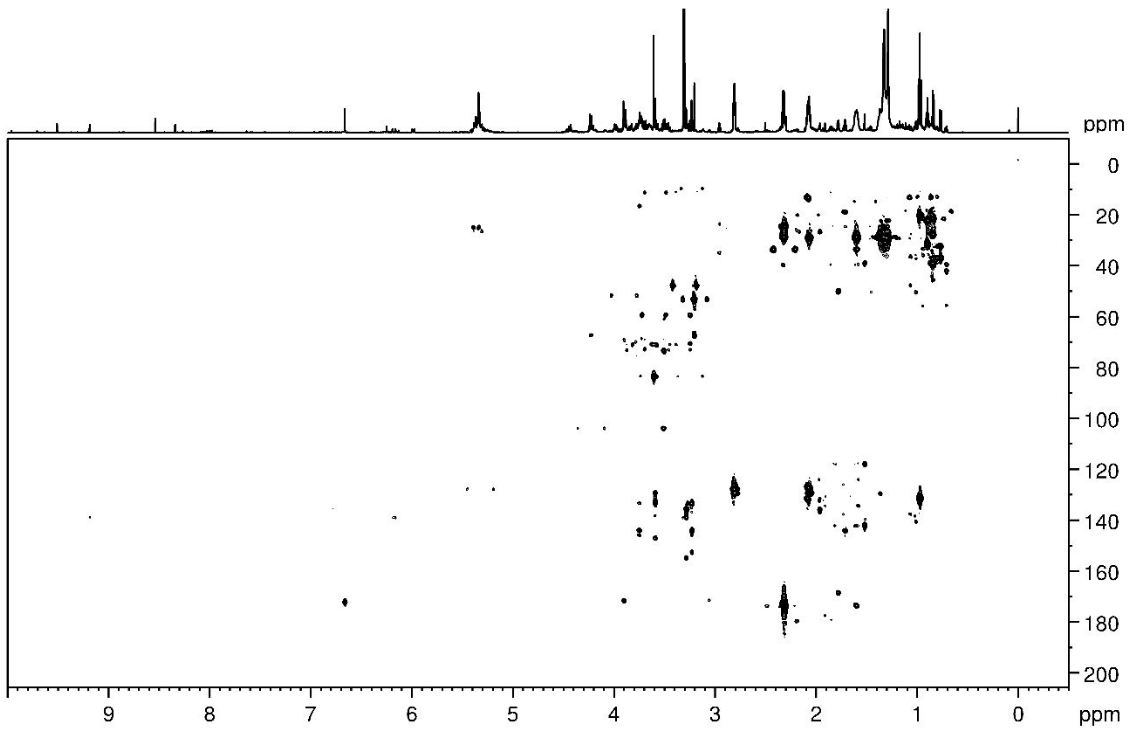

Figure S8. 2D NMR spectrum of HMBC ^1H-^{13}C (600 MHz) from CD_3OD extracts for soybean leaves from the control group.

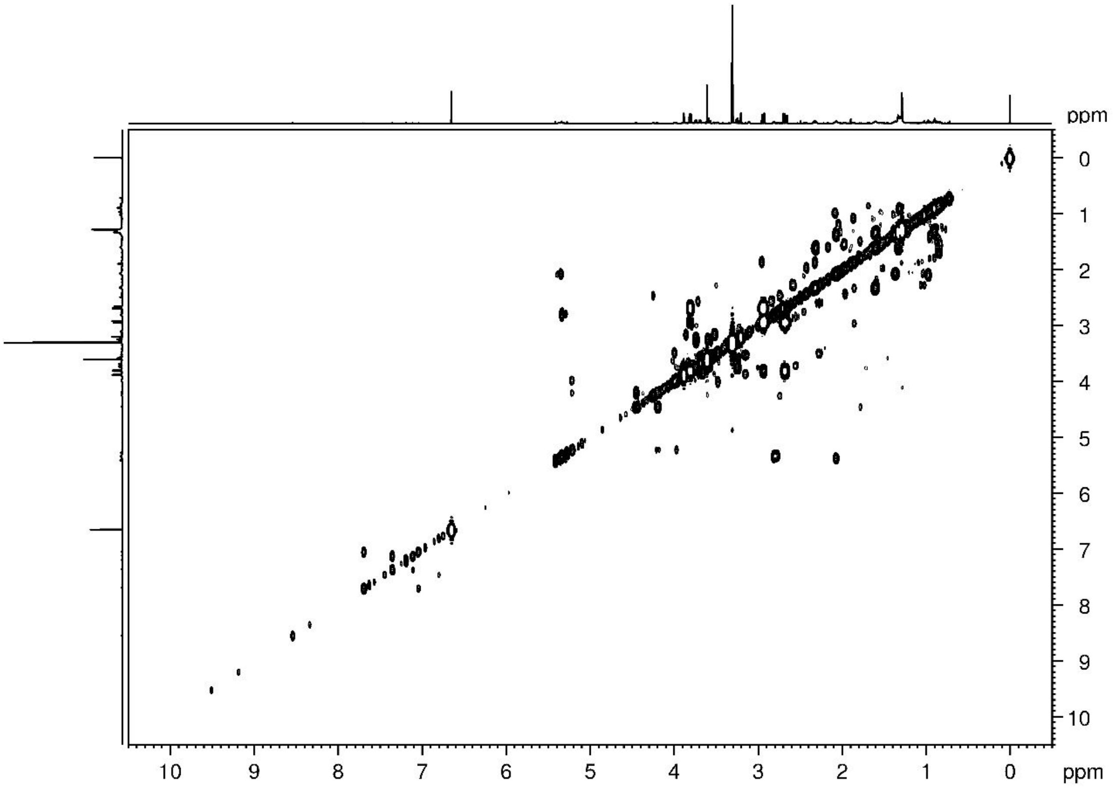

Figure S9. 2D NMR spectrum of COSY ^{1}H-^{1}H (600 MHz) from CD_3OD extracts for soybean stem from the control group.

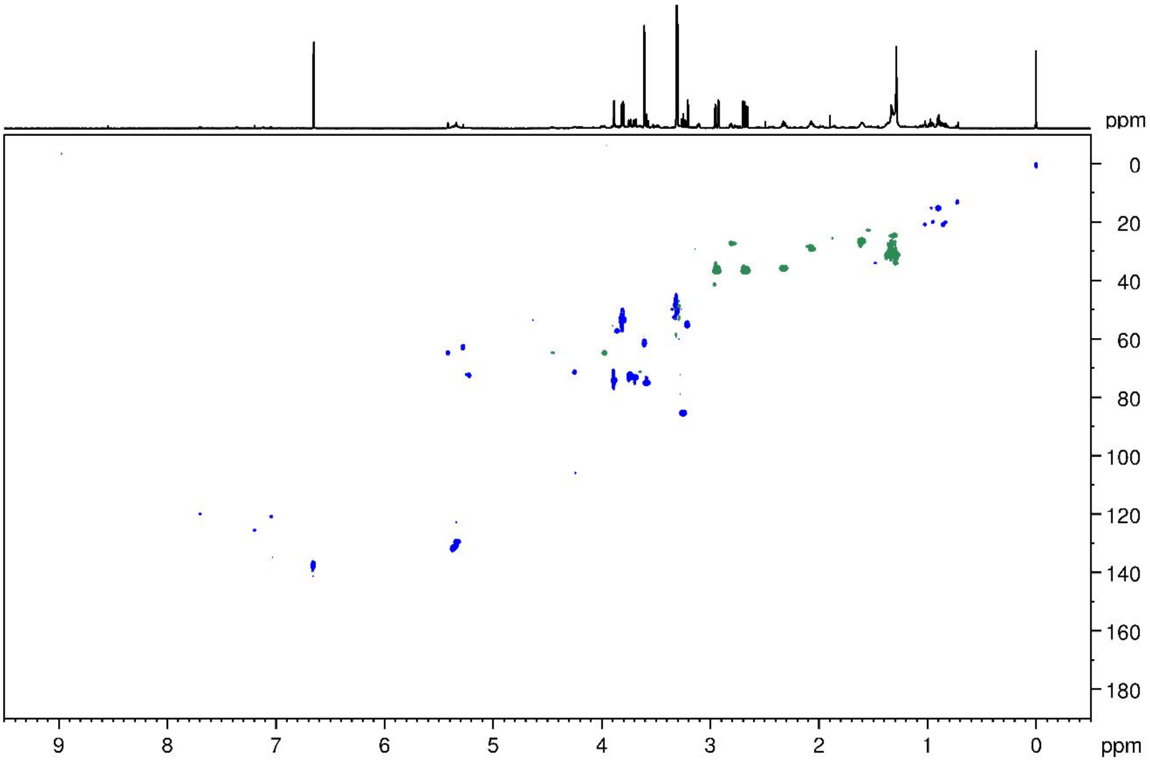

Figure S10. 2D NMR spectrum of multiplicity-edited HSQC ^{1}H-^{13}C (600 MHz) from CD_3OD extracts for soybean stem from the control group.

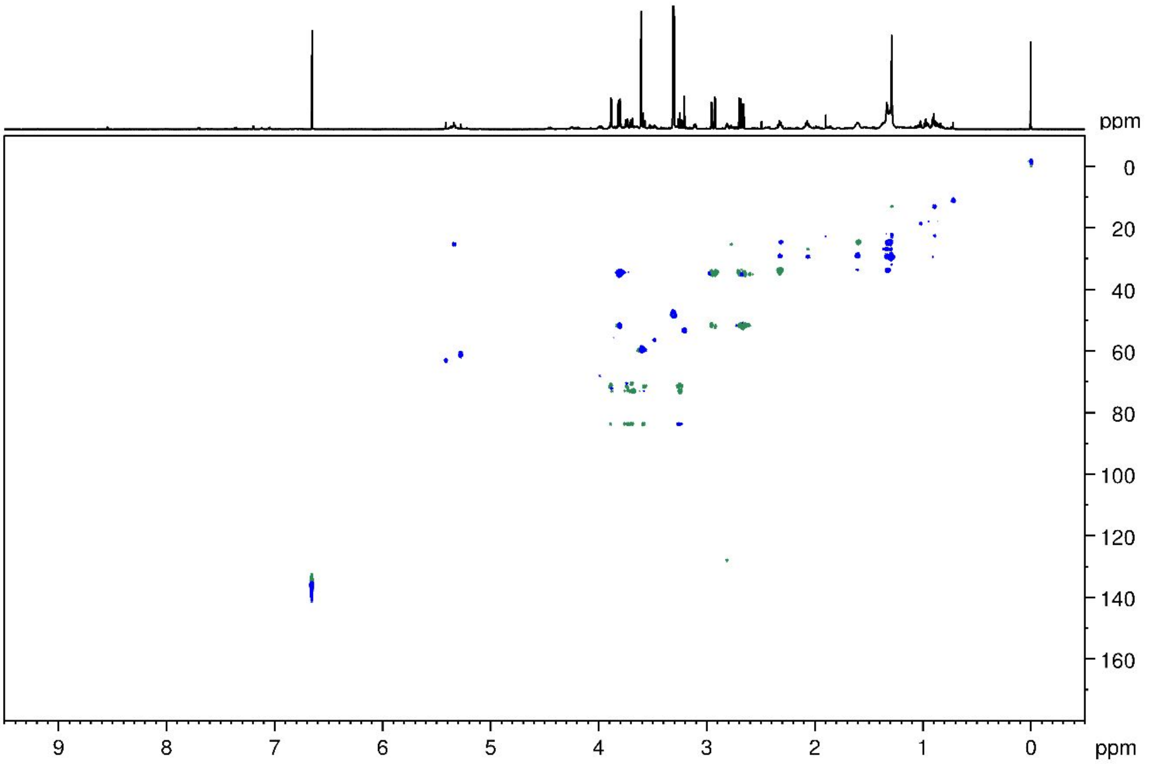

Figure S11. 2D NMR spectrum of HSQC-TOCSY (600 MHz) from CD_3OD extracts for soybean stem from the control group.

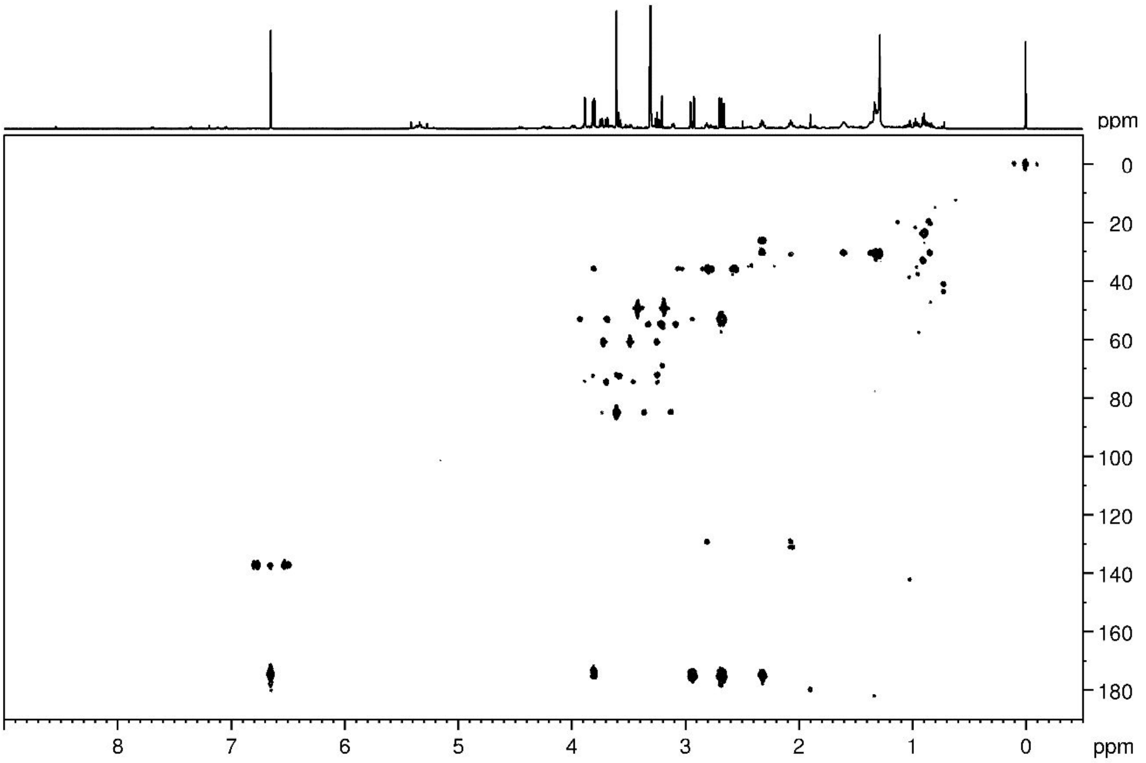

Figure S12. 2D NMR spectrum of HMBC ^1H-^{13}C (600 MHz) from CD_3OD extracts for soybean stem from the control group.

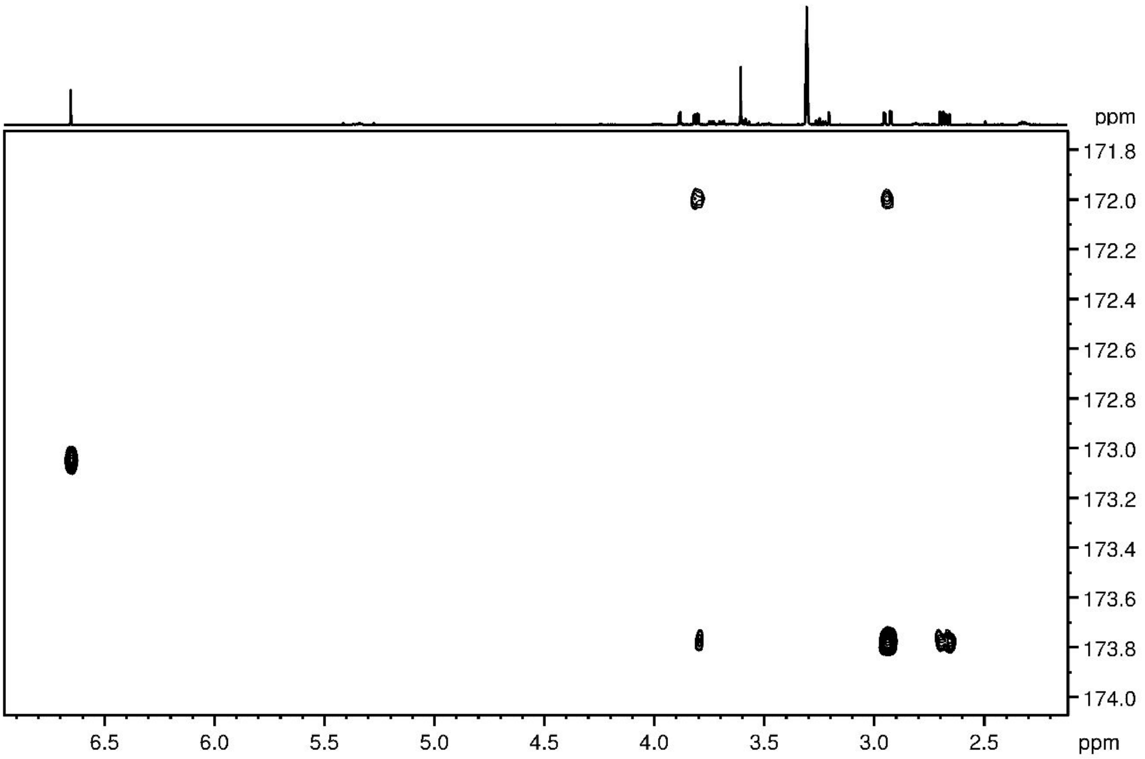

Figure S13. 2D NMR spectrum of selective HMBC ^{1}H-^{13}C (600 MHz) from CD_3OD extracts for soybean stem from the control group.

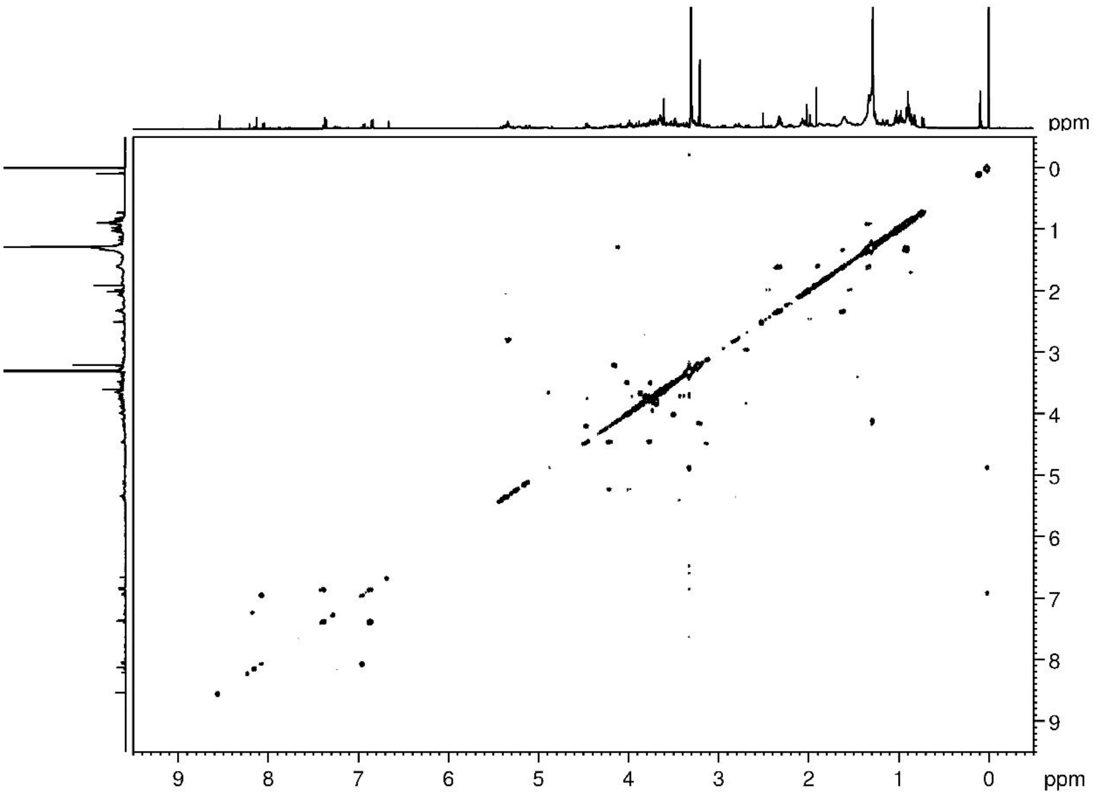

Figure S14. 2D NMR spectrum of COSY ^{1}H-^{1}H (600 MHz) from CD_3OD extracts for soybean root from the control group.

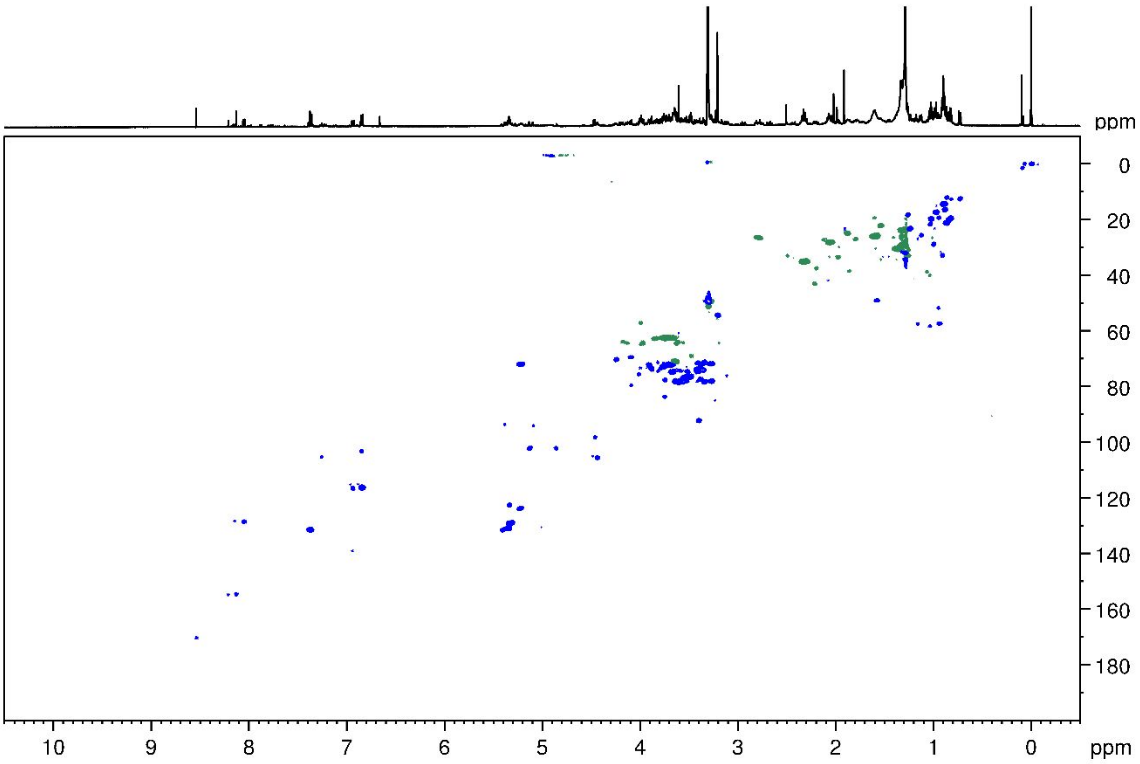

Figure S15. 2D NMR spectrum of multiplicity-edited HSQC ^{1}H-^{13}C (600 MHz) from CD_3OD extracts for soybean root from the control group.

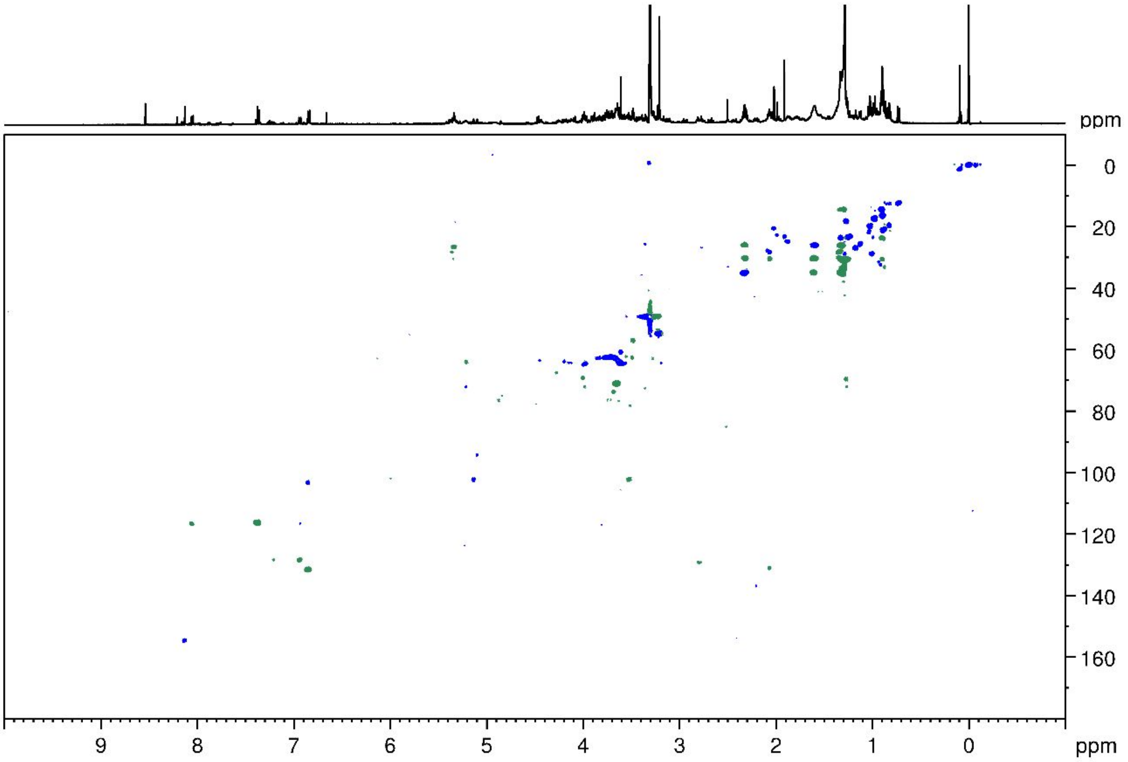

Figure S16. 2D NMR spectrum of HSQC-TOCSY (600 MHz) from CD_3OD extracts for soybean root from the control group.

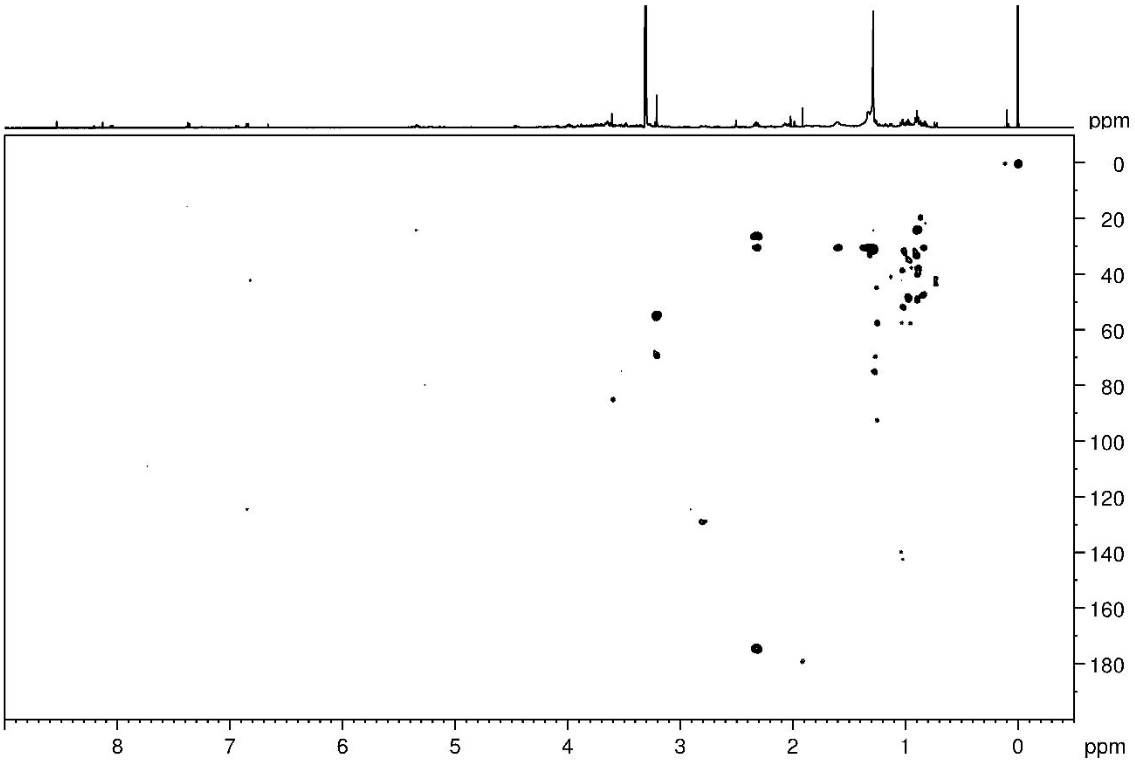

Figure S17. 2D NMR spectrum of selective HMBC ^{1}H-^{13}C (600 MHz) from CD_3OD extracts for soybean root from the control group.

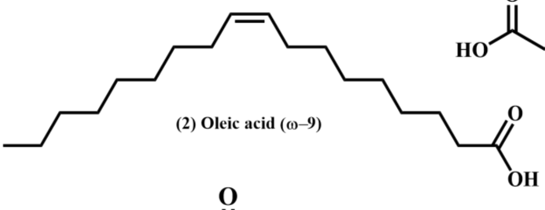

(2) Oleic acid (ω–9)

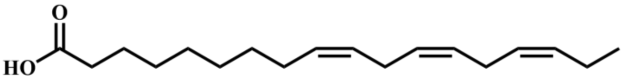

(4) α – Linolenic (ω–3)

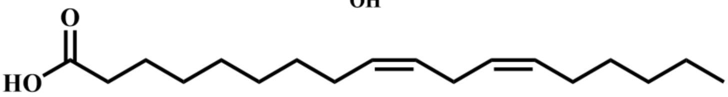

(3) Linoleic acid (ω–6)

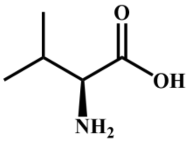

(7) Valine

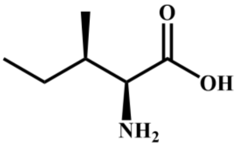

(8) Isoleucine

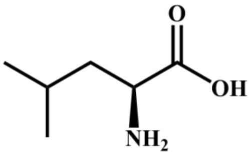

(9) Leucine

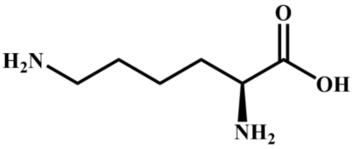

(10) Lysine

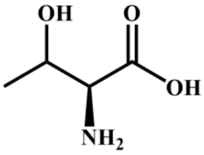

(11) Threonine

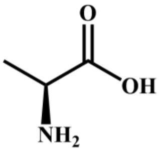

(12) Alanine

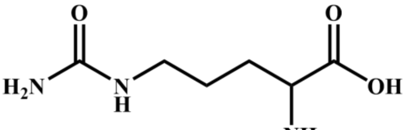

(13) Arginine

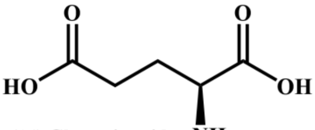

(14) Glutamic acid

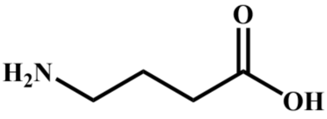

(15) γ-aminobutyrate (GABA)

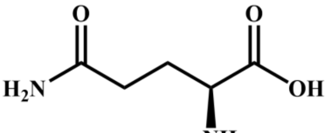

(16) Glutamine

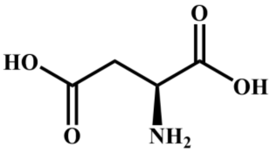

(17) Aspartic acid

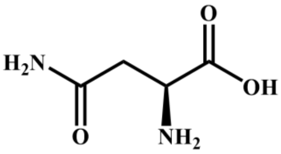

(18) Asparagine

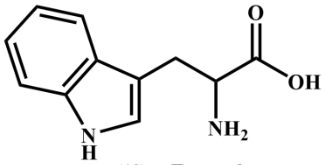

(19) Tryptophan

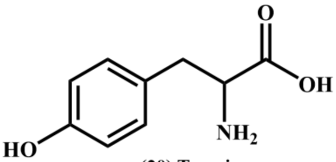

(20) Tyrosine

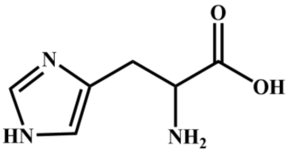

(21) Histidine

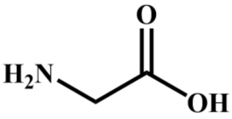

(22) Glycine

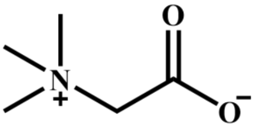

(23) Betaine

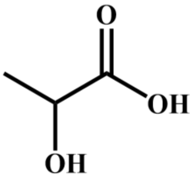

(24) Lactic acid

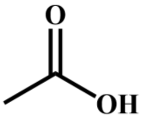

(25) Acetate

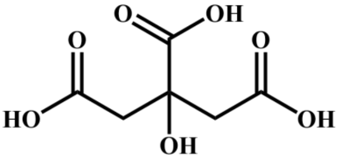

(26) Citric acid

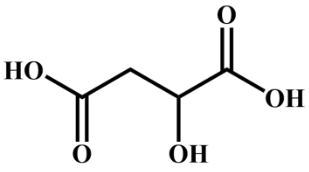

(27) Malic acid

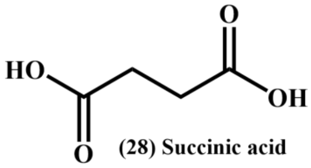

(28) Succinic acid

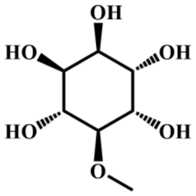

(29) Pinitol

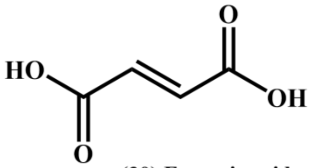

(30) Fumaric acid

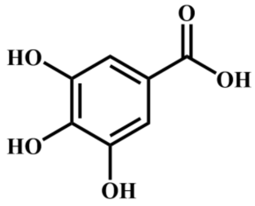

(31) Gallic acid

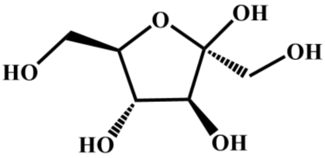

(32) Fructose

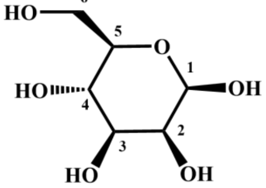

(33) β – Glucose

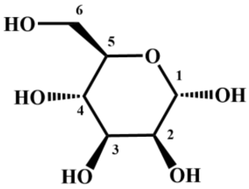

(34) α – Glucose

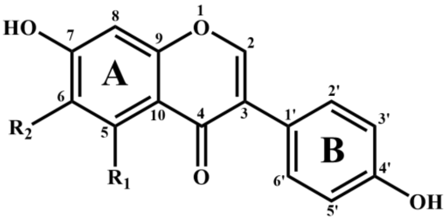

R_1	R_2	
OH	H	(**35**) Genistein
H	OCH_3	(**37**) Glycitein
H	H	(**40**) Daidzein

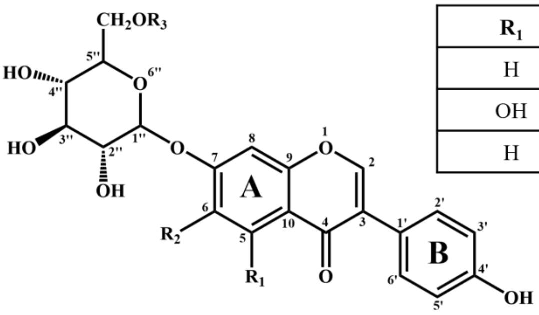

R_1	R_2	R_3	
H	OCH_3	H	(**36**) Glycitin
OH	H	H	(**38**) Genistin
H	H	H	(**39**) Daidzin

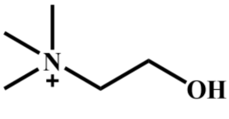

(41) Choline

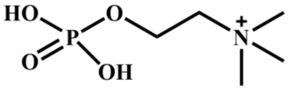

(42) Phosphorylcholine

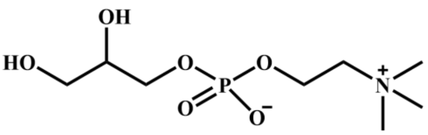

(43) Glycerophosphorylcholine

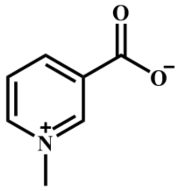

(44) Trigonelline

Figure S18. Chemical structures of assigned compounds.

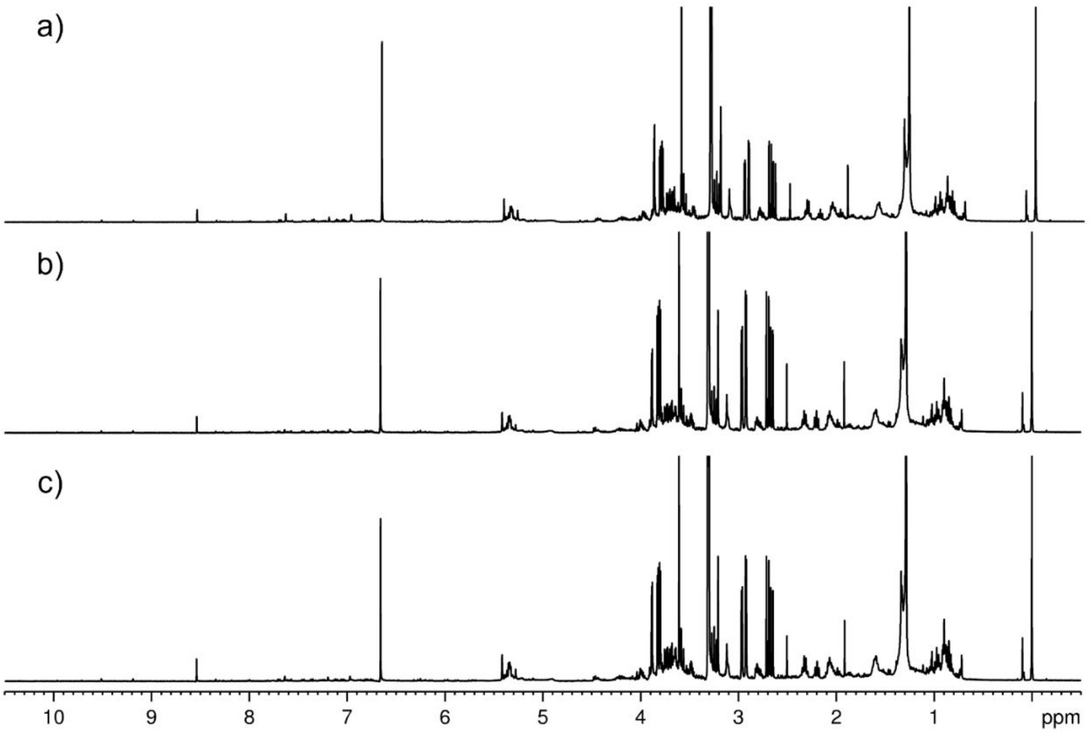

Figure S19. ^{1}H NMR spectra (400 MHz) with suppression of the residual water signal of CD_3OD extracts for Intact soybean stem from groups (**a**) T0 control group, (**b**) T1 AgNPs group and (**c**) T2 $AgNO_3$ group.

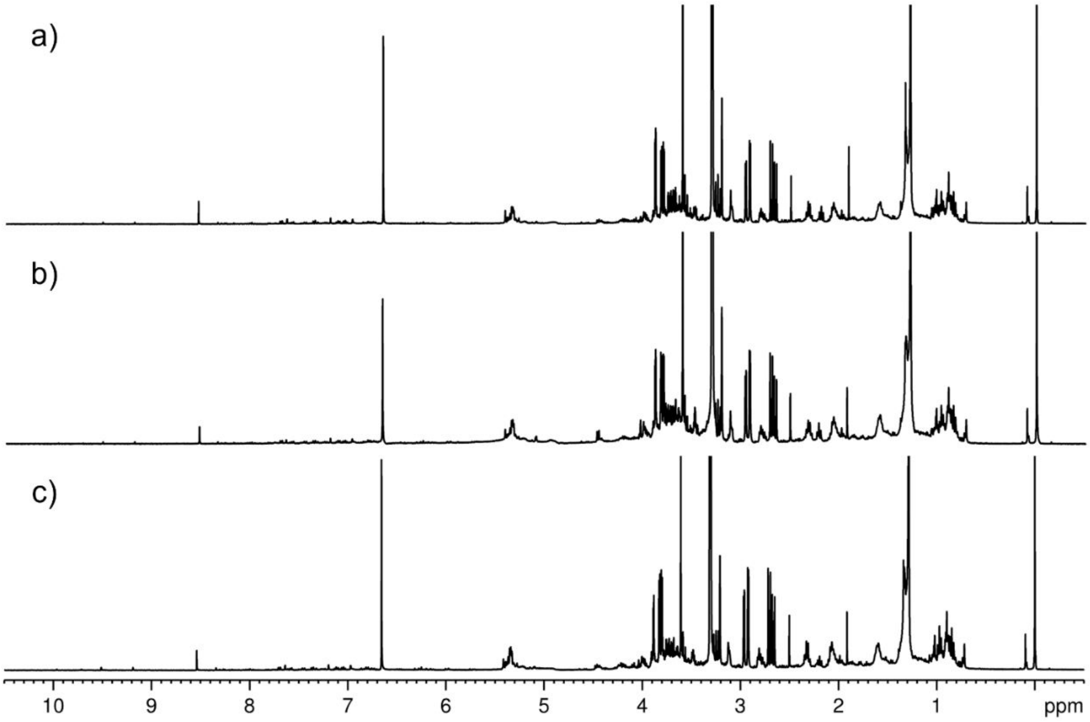

Figure S20. ^{1}H NMR spectra (400 MHz) with suppression of the residual water signal of CD_3OD extracts for RR soybean stem from groups (**a**) T0 control group, (**b**) T1 AgNPs group and (**c**) T2 $AgNO_3$ group.

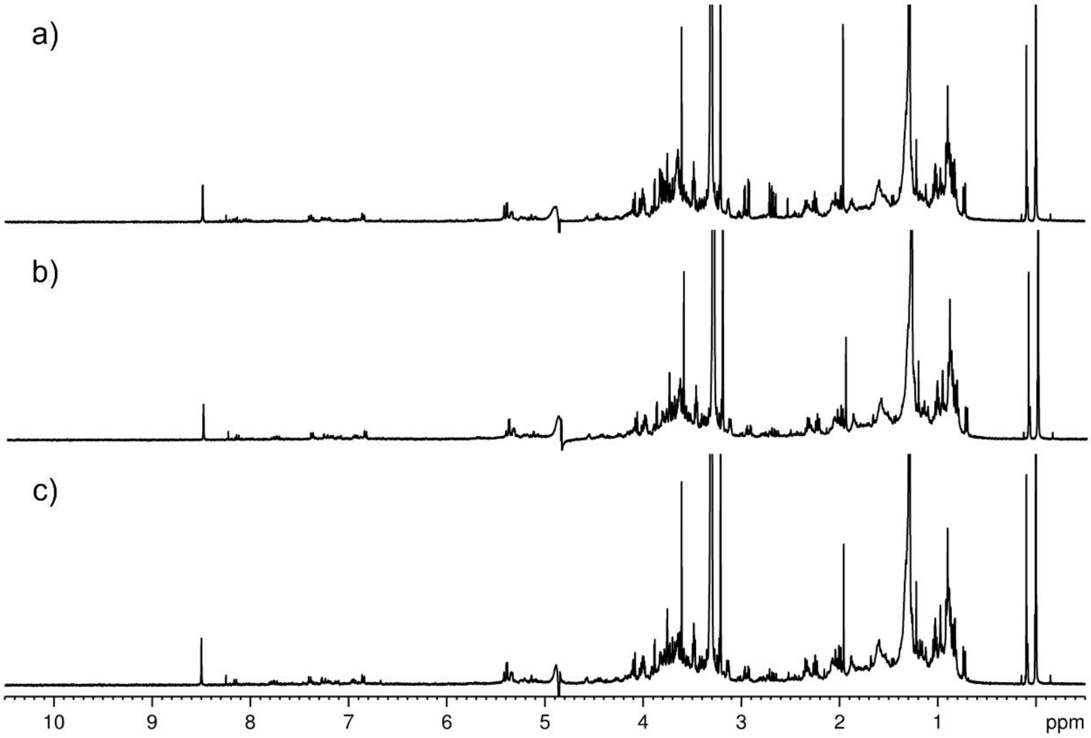

Figure S21. ^{1}H NMR spectra (400 MHz) with suppression of the residual water signal of CD_3OD extracts for Intact soybean root from groups (**a**) T0 control group, (**b**) T1 AgNPs group and (**c**) T2 $AgNO_3$ group.

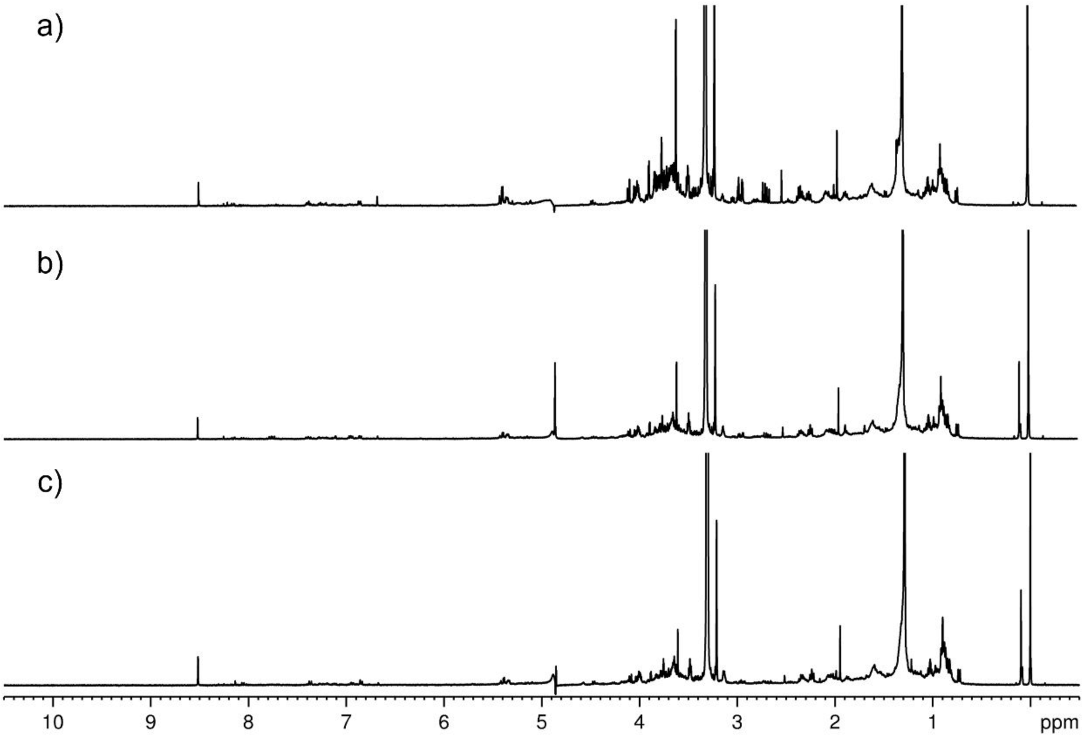

Figure S22. ^{1}H NMR spectra (400 MHz) with suppression of the residual water signal of CD_3OD extracts for RR soybean root from groups (**a**) T0 control group, (**b**) T1 AgNPs group and (**c**) T2 $AgNO_3$ group.
